# Design and validation of the Disaster Health Literacy Questionnaire for diabetes patients in Iran: a mixed-methods study

**DOI:** 10.1136/bmjopen-2025-106100

**Published:** 2025-11-24

**Authors:** Somayeh Panahi, Zahra Heidari, Maryam Heidarpour, Golrokh Atighechian, Hasan Ashrafi-rizi

**Affiliations:** 1 Department of Health Information Technology, School of Allied Medical Sciences, Golestan University of Medical Sciences, Gorgan, Iran; 2 Department of Biostatistics and Epidemiology, School of Health, Applied Physiology Research Center, Cardiovascular Research Institute, Isfahan University of Medical Sciences, Isfahan, Iran; 3 Isfahan Endocrine and Metabolism Research Center, Isfahan University of Medical Sciences, Isfahan, Iran; 4 Health Management and Economics Research Center, Isfahan University of Medical Sciences, Isfahan, Iran; 5 Medical Library and Information Science Department, Health InformationTechnology Research Center, Isfahan University of Medical Sciences, Isfahan, Iran

**Keywords:** Trauma, Health Literacy, Chronic Disease, Surveys and Questionnaires, Health Education

## Abstract

**Abstract:**

**Objectives:**

To develop and psychometrically evaluate a multidimensional Disaster Health Literacy Questionnaire (DHLQ) for diabetic patients in Iran, using advanced item response theory approaches. The questionnaire was designed in the Persian (Farsi) language.

**Design:**

A sequential mixed-methods study incorporating qualitative (scoping review and interviews) and quantitative (psychometric validation) phases.

**Setting:**

Diabetes clinics and healthcare centres across Iran (2022–2023).

**Participants:**

The study enrolled 570 patients with diabetes (56% female, mean age 45.57±16.33 years) for quantitative validation; 15 experts and 15 patients for qualitative validation.

**Outcome measures:**

The psychometric properties evaluated included content validity (using content validity ratio (CVR) and content validity index (CVI)), construct validity (assessed via multidimensional item response theory (MIRT)), and reliability (measured by Cronbach’s alpha and test-retest Kappa). Additionally, item parameters (multidimensional difficulty (MDIFF) and multidimensional discrimination (MDISC)) and model fit indices (RMSEA, CFI and TLI) were examined.

**Results:**

The final 30-item DHLQ demonstrated excellent content validity (scale-level CVI=1; item-level CVI>0.79; CVR>0.49). Cronbach’s alpha for the total scale was 0.606; test-retest reliability showed significant agreement (Kappa=0.35–1, p<0.05). MIRT confirmed a three-factor structure: Disaster Perception Risk (14 items), Medication-Nutritional Literacy (11 items), and Self-help and Emergency Literacy (five items). Model fit was excellent (RMSEA=0.016, CFI=0.96, TLI=0.95). Item analysis revealed that 73% of items had moderate-to-high discrimination (MDISC ≥0.65), and 83% had medium-to-low difficulty (MDIFF <0.5).

**Conclusion:**

The DHLQ is a rigorously validated tool for assessing disaster health literacy in diabetic populations. Its multidimensional structure and strong psychometric properties support its use in clinical and emergency planning contexts to identify literacy gaps and tailor interventions.

**STRENGTHS AND LIMITATIONS OF THIS STUDY**

- The study used a sequential mixed-methods design to inform questionnaire development.
- Construct validity was rigorously assessed using multidimensional item response theory.
- The sample size for quantitative validation (n=570) provided robust data for psychometric analysis.
- The findings of the study may have limited generalisability due to its specific cultural and geographic focus.
- The reliance on self-reported data poses a potential risk of social desirability bias.

## Introduction

 Diabetes is among the top six chronic diseases globally, both in terms of prevalence and mortality. Current reports indicate that by 2050, more than one billion people are expected to be affected by diabetes.^1^ Research indicates a global increase in diabetes prevalence, highlighting the need for enhanced prevention and management strategies to address serious complications. The annual cost of diabetes is approximately $673 billion, or about 12% of national healthcare budgets allocated for its treatment.^2 3^ Effective management is linked to behavioural aspects, with psychological and social factors being crucial.^4^ Some experts view diabetes as a behavioural issue manageable through self-care. Consequently, health literacy has emerged as a vital skill for diabetic patients, enabling informed health decisions in challenging circumstances.^5 6^ Disaster health literacy (DHL) extends this concept, encompassing the skills and knowledge that enable individuals and communities to effectively mitigate health risks, respond to emergencies and facilitate recovery.^7^ Ultimately, its core purpose is to empower people to make critical, informed health decisions under extreme pressure, thereby reducing mortality, preventing secondary health disasters and enhancing overall community resilience in the face of crises.^7^ This includes the ability to understand early warning messages, adhere to evacuation orders, access essential healthcare services amid chaos, and practise preventive measures such as hygiene and water purification to prevent disease outbreaks in disaster-affected communities.^8^

Disruptions to the healthcare system and damage to pharmaceutical storage have severely compromised medication supply chains, leading to critical shortages. Furthermore, volunteer organisations often lack specialised resources for diabetic patients and strategies for maintaining proper medication storage conditions. Given the disruptions in treatment access during crises, enhancing health literacy for diabetic patients is crucial for reducing disaster impacts. Comprehensive planning and strategic initiatives can improve their preparedness and health outcomes during emergencies.^9^,^11^ A significant challenge in health literacy is the vulnerability of diabetic patients to both natural and man-made disasters, which threaten their health. Statistics show an increase in crises affecting these patients. Research from Iran, a context of elevated disaster risk common to many low and middle-income countries, reveals that approximately two-thirds of patients with diabetes or chronic obstructive pulmonary disease lack sufficient disaster preparedness knowledge, highlighting a critical vulnerability that demands targeted intervention.^12^

With the increasing frequency of natural disasters like earthquakes and floods, bolstering DHL for at-risk populations, particularly diabetics, is imperative. While educational programmes have focused on healthcare providers, the self-care needs of diabetic patients during disasters remain under-addressed. To tackle this issue, the first step is to develop a valid tool to assess DHL among diabetic patients. Currently, no reliable instrument exists for this purpose. This study aims to create and validate a DHL Questionnaire (DHLQ) tailored for diabetic individuals, which is expected to enhance their preparedness and inform future research in this critical area.

## Methods

### Data analytic plan

This study used a mixed-methods approach, integrating both confirmatory and exploratory research to design and psychometrically assess the DHLQ, which was designed in Farsi, specifically targeting diabetic patients. The investigation was conducted in two phases: qualitative and quantitative. The qualitative findings informed the development of the initial item pool, which was then quantitatively tested for psychometric properties. The qualitative phase involved a comprehensive scoping review and semi-structured interviews conducted between 2022 and 2023, with detailed findings published in relevant articles.^8 13^ The qualitative analysis yielded five key themes: disaster perception, risk literacy, medication literacy, resilience literacy, nutrition literacy and self-help literacy, along with associated subcategories. Based on the qualitative results, an initial pool of test items (multiple choice questions) was generated and subsequently reviewed by the research team. After revisions, the questionnaire proceeded to the quantitative phase, comprising a total of 54 items.

### Validity and reliability assessment

This section was reorganised into four sub-sections: content validity, face validity, reliability and construct validity.

### Content validity

Content validity refers to the degree to which the content of a measurement tool aligns with the research objectives.^14^ To assess content validity, both qualitative and quantitative methods were used. In the qualitative review, the questionnaire was provided to 15 experts: 5 endocrinologists, 4 disaster response specialists with at least 5 years of experience, 4 nurses and 2 medical librarians familiar with the field of DHL. These experts were asked to provide feedback regarding the alignment of the content of the questionnaire with the research objectives, suggesting necessary revisions to simplify, clarify and make the questions more relevant, and to remove any redundant concepts. Based on their feedback, the necessary revisions were made, and the final version of the questionnaire was approved by all experts. For the quantitative assessment of content validity, both the content validity ratio (CVR) and content validity index (CVI) were used.

#### Content validity ratio

For this, 15 experts were asked to rate each item on a 3-point scale: ‘Essential’, ‘Important but not essential’, and ‘Not essential’. Given that the panel consisted of 15 experts, the minimum acceptable value for the CVR, according to the Lawshe table, was considered to be 49%.^15^

#### Content validity index

To calculate the CVI, the questionnaire was provided to the same 15 experts. They were asked to rate the relevance, simplicity and clarity of the items on a scale from 1 to 4. For calculating the item-level CVI (I-CVI), the number of experts who rated an item 3 or 4 was divided by the total number of experts. Items with an I-CVI value greater than 0.79 were accepted, those between 0.7 and 0.79 were revised, and those below 0.69 were deleted. In the next step, based on the average scores for the CVI of all items, the scale-level CVI using the average method (S-CVI/average) was calculated for the entire tool. Polit and Beck recommended an overall CVI score of 0.9 or higher for acceptance.^16 17^ When disagreements among experts arose, they were resolved through discussion and consensus within the expert panel. Several items were modified slightly based on their feedback to improve clarity and relevance.^16^

### Face validity

Face validity refers to the degree to which the items of a tool appear to be relevant and aligned with the intended purpose of the tool. It was assessed both qualitatively and quantitatively. In the qualitative phase, the questionnaire items were reviewed by the target population (15 diabetic patients) to assess the level of difficulty, relevance and any potential ambiguities. This review led to the simplification and clarification of items, making them more understandable and relevant.

In the quantitative phase, the participants were asked to rate each item based on its importance. The item impact score was then calculated. For this purpose, a 5-point Likert scale (1–5) was used for each item in the questionnaire, and the target group was asked to rate each question according to the following options: ‘Very Important’, ‘Somewhat Important’, ‘Moderately Important’, ‘Slightly Important’, and ‘Not Important at All’. The item impact score was calculated using the following formula:

Impact Score = Frequency (%) × Importance

Where the frequency percentage of participants who rated the item as important (ratings of 4 and 5) is multiplied by the average importance score of the item (mean rating). If the impact score was greater than 1.5, the item was considered suitable for further analysis and was retained.^18^

### Reliability

In this study, Cronbach’s alpha coefficient was used to determine the reliability and internal consistency of the questionnaire. A Cronbach’s alpha value above 0.90 was considered excellent, between 0.90 and 0.80 was considered good, between 0.80 and 0.70 was acceptable, between 0.70 and 0.60 was considered questionable, between 0.60 and 0.50 was considered weak, and below 0.50 was considered unacceptable. The Cronbach’s alpha for the entire questionnaire, as well as changes in alpha when each item was removed, was calculated.^19^

To determine the stability of the results, a test-retest method was used. For this purpose, 30 diabetic patients completed the questionnaire at two different time points, with a 2-week interval between the two tests. The scores from the two time points were then compared using the Kappa coefficient. The Kappa coefficient ranges from −1 to +1, with values closer to +1 indicating greater agreement between the scales, and values closer to −1 indicating less agreement.^18 20^

### Participants

Demographic characteristics of the participants in the content validity, face validity, post-test and final sample stages are presented in table 1.

**Table 1:** Characteristics of participants for content/face validity

Group	Total (N)	Age range (years)	Professional/ education level/n	With diabetes	Type of diabetes		Key characteristics and experience
					1	2	
Expert panel	15	36–61	Endocrinologists (5)	–	–	–	Prior experience in disaster and rescue teams Research/executive work experience in diabetes or personal experience with diabetes (✓ for 5 experts)
			Disaster specialists (4)	2	–	2	
			Nurses (4)	1	1	–	
			Medical Librarians (2)	2	–	2	
Patient panel	15	18–58	Middle school education (4)	4	3	1	Personal experience with at least one crisis Have active medical records at the hospital
			Diploma	4	4	–	
			Bachelor	5	2	3	
			PhD	2	–	2	
Test-retest panel	30	18–65	Middle school education	4	3	1	Possessing basic literacy skills Stable health condition during the test interval Willingness to participate in both phases of the study
			Diploma	9	4	5	
			Bachelor	11	7	4	
			PhD	6	1	5	
Main validation sample	570	18–82	Middle school education (187)	187	83	104	Confirmed diabetes diagnosis (type 1/2), Accessible medical records Convenience sampling from teaching hospitals affiliated with Isfahan University of Medical Sciences
			Diploma	78	51	27	
			Bachelor	245	117	128	
			PhD	60	18	42	
BMI status	570	18–82	Slim		6		
			Normal		234		
			Overweight		276		
			Obese		54		

BMI, body mass index.

The sample size of 570 participants was determined based on commonly recommended guidelines for both factor analysis and item response theory (IRT) modelling. For exploratory and confirmatory factor analysis (EFA and CFA), a sample of at least 5–10 participants per item is generally suggested; with the initial 37-item questionnaire, our sample meets this criterion (37 × 10 = 370). For IRT and multidimensional IRT (MIRT) analyses, larger samples improve parameter estimation stability, and previous studies suggest that 500–600 participants are sufficient for dichotomous item calibration in instruments of similar length. After item reduction, the final 30-item instrument still maintains an adequate subject-to-item ratio, ensuring robust estimation of item parameters and model fit. In this study, the 570 participants were selected using a convenience sampling approach (available participants) . Questionnaires were distributed in teaching hospitals affiliated with Isfahan University of Medical Sciences located in Isfahan city.

### Construct validity

#### Item response theory analysis

The construct validity of the developed tool was evaluated through EFA and CFA using data from 570 completed questionnaires. Considering the dichotomous nature of the responses (correct or incorrect), the data in this study were analysed using logistic IRT^21^ models.^20^ IRT models are mathematical functions that determine the probability of a correct response to a question based on examinee parameters (ability) and item parameters (difficulty, discrimination and guessing).^22^

Initially, 37 items of the questionnaire were analysed using a two-parameter logistic IRT model. Unidimensionality testing and CFA were performed, and item parameters such as difficulty and discrimination power were estimated. It is important to note that the implementation of IRT requires two basic assumptions: unidimensionality and local independence of items.^23^ The unidimensionality assumption posits that the items of a test measure only one latent ability, regardless of the cognitive or personal characteristics of the individuals. Another key assumption, local independence, can be defined as the lack of a significant relationship between the responses to items.^23 24^ If the data structure is not unidimensional, the psychometric indices estimated using unidimensional IRT, particularly the discrimination index, will lack accuracy.

Thus, in parallel with the implementation of IRT, unidimensionality testing and CFA were conducted to determine whether the data exhibit a unidimensional structure. The CFA results were assessed using three fit indices: RMSEA (with a good fit threshold of less than 0.06 and an acceptable fit threshold of less than 0.08), CFI and TLI (with a good fit threshold above 0.95 and an acceptable fit threshold above 0.90). Given the rejection of the unidimensionality assumption, we moved toward multidimensional modelling to better reflect the complex structure of the construct.

#### Exploratory factor analysis

To examine the potential latent dimensions of the developed tool, EFA (ranging from 2-factor to 6-factor solutions) was conducted using principal component analysis to calculate factor loadings and Geomin rotation for factor extraction. The adequacy and suitability of the data for conducting this analysis were assessed using the Kaiser-Meyer-Olkin (KMO) index (KMO=0.683) and Bartlett’s test of sphericity (p<0.001). Significant factor loadings were used to assign items to each dimension of the questionnaire. The resulting 3-factor structure was confirmed by the researchers and used as the initial input for the MIRT model.

#### Multidimensional item response theory analysis

Finally, the factor structure of the developed tool was evaluated using the compensatory MIRT model,^25^ which included the results of the EFA and CFA as well as item difficulty and discrimination. From these parameters, two key indices were derived to interpret the items: multidimensional discrimination (MDISC) and multidimensional difficulty (MDIFF).

According to the classification criteria adopted from Hasmy (2014) and Baker (2001),^26 27^ the values of MDISC and MDIFF can be interpreted as shown in table 2.

**Table 2:** MDISC classification (left) and MDIFF classification (right)

Criteria	Description	Criteria	Description
MDISC ≥ 1.7	Very high	MDIFF ≥ 2	Very hard
1.35 ≤ MDISC < 1.7	High	0.5 ≤ MDIFF < 2	Hard
0.65 ≤ MDISC < 1.35	Moderator	−0.5 ≤ MDIFF < 0.5	Medium
0.35 ≤ MDISC < 0.65	Low	−2 ≤ MDIFF < −0.5	Easy
MDISC < 0.35	Very low	MDIFF < −2	Very easy

MDIFF, multidimensional difficulty; MDISC, multidimensional discrimination.

The MIRT approach was selected because it allows simultaneous estimation of multiple latent traits, which aligns with the multidimensional nature of the DHLQ (eg, medication, resilience and nutrition literacy). This approach provides more accurate item-level estimates and supports stronger evidence for construct validity compared with traditional unidimensional models.

In simple terms, this model estimates how well each question differentiates between respondents with varying levels of DHL across several dimensions, and how difficult each question is relative to those dimensions.

Exploratory factor analysis was conducted using Mplus (Version 8.11; Muthén & Muthén, Los Angeles, CA, USA). Item Response Theory (IRT) and Multidimensional IRT (MIRT) analyses were performed using the 'mirt' package within the R statistical computing environment (Version 4.2.3; R Foundation for Statistical Computing, Vienna, Austria).

Exploratory factor analysis was conducted using Mplus (Version 8.11; Muthén & Muthén, Los Angeles, CA, USA)., while IRT and MIRT analyses were performed using the ‘Mirt’ package in the free R software version. *Exploratory factor analysis was conducted using Mplus (Version 8.11; Muthén & Muthén, Los Angeles, CA, USA). Item Response Theory (IRT) and Multidimensional IRT (MIRT) analyses were performed using the 'mirt' package. within the R statistical computing environment (Version 4.2.3; R Foundation for Statistical Computing, Vienna, Austria).*

## Results

### Initial item pool development

In this study, based on the qualitative phase results (domain review and follow-up interviews) and the aggregation of the opinions of the research team, five dimensions of health literacy were identified: ‘Disaster Risk Literacy, Medication Literacy, Nutrition Literacy, Resilience Literacy and Self-help Literacy’, which were selected as measurable variables and constructs for the questionnaire. The findings have been presented in the published articles.^8 13^ Accordingly, the research team was able to design an initial pool of questionnaire items in a multiple choice question format. During several joint sessions, overlapping questions were merged by the research team. Additionally, questions with a high level of specialisation were removed, and as a result, 54 items remained in the first phase of the refinement process.

### Item reduction

The initial questionnaire with 54 test questions was designed, and these items were then subjected to psychometric analysis. In the content validity assessment, the proposed changes by specialists during the qualitative phase were applied to the appearance of the questions. The most significant changes based on feedback by specialists included modifications to the structure and wording, merging, revising and removing certain items. Ultimately, after merging and removing 5 questions (out of 54), 49 items remained. In the quantitative phase of content validity, the number of items was reduced to 37. Twelve items had a CVR below 0.49 and were removed. Based on the judgement of the researcher, only one item with a CVR of 0.47 was retained due to its minimal deviation from the threshold. According to the opinions of 15 specialists, the calculated CVI for these 37 items was greater than 0.79. Therefore, all 37 items were retained in this phase. Additionally, the average CVI (S-CVI) for the entire questionnaire was calculated to be 1.

### Face validity

In the qualitative face validity phase, minor editorial changes were made based on feedback from 15 patients, and all 37 items were approved. In the quantitative phase, no items had an impact score below 1.5, so all 37 items were retained for further analysis.

### Reliability

The questionnaire was administered experimentally to 30 diabetic patients, and the Cronbach’s alpha coefficient for the 37-item questionnaire was calculated to be 0.57, which falls within the low range. On examining changes in alpha for deletion of each item, it was found that removing questions 5, 19, 21, 24, 35 and 36 resulted in an increase in alpha. The same 30 patients completed the questionnaire again after a 2-week interval. The Kappa agreement coefficient values for the 37 questionnaire items in the test-retest scenario were statistically significant at the 0.05 level, indicating meaningful agreement and test stability. Online supplemental file 1 presents the values of CVI, CVR, item impact scores and Kappa agreement coefficients for the 37 questionnaire items, and Cronbach’s alpha for deletion of each item.

### Descriptive statistics and associations with demographic variables

Prior to the main IRT analysis, descriptive statistics and associations with demographic variables were examined. To examine the factor structure, the 37-item questionnaire was completed by 570 diabetic patients. Of the participants, 322 (56%) were female. The mean age of the participants was 45.57±16.33 years (range: 18–82 years). Regarding diabetes-related characteristics, 27% of participants had type 1 diabetes and 73% had type 2 diabetes. Approximately 28% reported having at least one comorbid condition, and 9.5% were classified as obese based on body mass index data. In terms of education level, 46.5% of participants had completed high school or lower, while 53.5% held a university degree. The majority of participants (62%) reported having experienced at least one hazardous or disaster-related event in the past. The mean duration of diabetes among respondents was 9.47±7 years (range: 1–50 years). Descriptive statistics, including the number of respondents, mean, standard deviation (SD), and minimum and maximum scores, were calculated for the questionnaire scores across all demographic subgroups.

The normality of the total scores, age and diabetes duration was assessed using Q–Q plots, histograms and box plots. The results showed no significant deviation from a normal distribution (skewness for total score = –0.604). Pearson correlation analysis indicated that neither age (r = –0.060, p=0.154) nor diabetes duration (r=0.030, p=0.468) was significantly correlated with the total DHL score, suggesting that these variables did not have a meaningful linear relationship with DHL. Based on the independent t-test and one-way analysis of variance results, there were no significant differences in the mean scores between women and men (p=0.968), type of diabetes (p=0.855), comorbidities (p=0.208) or obesity status (p=0.070). However, significant differences were found between the mean scores of different educational levels (p<0.001) and between those who had or had not experienced an incident (p=0.001; table 3). Therefore, individual capabilities appear to impact the scores, supporting the use of IRT in analysing the questionnaire.

**Table 3:** Comparison of mean total scores of the questionnaire between different subgroups

Number of participants	Mean	SD	Minimum	Maximum	P value
322 (female)	22.48	4.08	9	30	0.968
248 (male)	22.49	4.1	12	31	
265	21.77	4.13	9	30	< 0.001
305	23.11	3.95	11	31	
155	22.54	4.07	12	29	0.855
415	22.47	4.1	9	31	
408	22.62	3.81	11	31	0.208
162	22.14	4.7	9	31	
6	22.33	3.72	16	26	0.07
234	22.47	3.8	9	31	
276	22.76	4.07	11	31	
54	21.15	5.14	10	30	
215	23.21	3.22	11	30	0.001
355	22.04	4.48	9	31	
570	22.48	4.09	9	31	

### Construct validity

#### Item response theory analysis

A two-parameter logistic IRT^21^ model was first estimated for the 37 questionnaire items. The CFA results indicated a poor fit for a unidimensional structure (RMSEA=0.04, CFI=0.627, TLI=0.604; table 4). Six items were sequentially removed based on low item–total correlations, improvements in Cronbach’s alpha and inappropriate IRT parameters (difficulty and discrimination), supported by expert judgement (online supplemental file 2). This iterative process resulted in a reduced 31-item model. Although this model demonstrated improved fit indices (RMSEA=0.04, CFI=0.67, TLI=0.65) and lower AIC and BIC values, the overall model fit still did not support a strictly unidimensional structure. Parallel analysis results (table 4) confirmed this conclusion for both models. In the 37-item IRT model, the second observed eigenvalue (2.20) exceeded the average simulated eigenvalue (1.22; p=0.0099), indicating the rejection of unidimensionality. A similar pattern was observed for the 31-item model, where the second eigenvalue (1.95) remained higher than the simulated average (1.09; p=0.0099). These results consistently demonstrated that the data did not conform to a single underlying latent dimension.

**Table 4:** Results of the parallel analysis for testing unidimensionality

Model	Statistic	Observed data	Average from 100 Monte Carlo samples	P value	Interpretation
IRT (37 items)	Second eigenvalue	**2.195**	**1.225**	**0.0099**	Reject unidimensionality assumption
IRT (31 items)	Second eigenvalue	1.947	1.091	**0.0099**	Reject unidimensionality assumption

Further examination of two-way margins revealed significant χ^2^ residuals ((O–E)²/E>3.5) among several item pairs in both models—for example, items 25–30, 26–30 and 1–30 in the 37-item model, and items 21–26, 1–26 and 1–21 in the 31-item model—indicating local dependence violations. Considering the evidence from the CFA, parallel analysis and local independence tests, the use of an MIRT model was deemed necessary to better capture the underlying structure and inter-item relationships within the questionnaire.

#### Exploratory factor analysis

Given the repeated rejection of the unidimensionality assumption, exploratory factor analyses with two-factor to six-factor models were performed to identify the latent dimensions of the developed instrument. The data were deemed adequate for factor analysis, as indicated by a KMO value of 0.683 and a significant Bartlett’s test of sphericity (p<0.001). Following a thorough review of the results by the research team and subject-matter experts, the three-factor model was confirmed as the most interpretable and statistically robust structure. Items showing significant factor loadings were assigned to their respective dimensions (table 5), whereas item 9, which failed to load significantly on any factor, was removed. Following this adjustment, the internal consistency (Cronbach’s alpha) of the 30-item instrument improved to 0.61.

**Table 5:** Three-dimensional exploratory analysis

Questions	Question number in the analysis	Factor 1	Factor 2	Factor 3
q1	U1	−0.146	0.235*	−0.404*
q2	U2	0.440*	0.08	0.013
q3	U3	0.483*	−0.117	−0.126
q4	U4	0.299*	−0.179*	0.121
q6	U5	0.168*	0.418*	0.119
q7	U6	0.132	0.170*	−0.336*
q8	U7	0.413*	0.136	0.098
q9	**U8**	**0.133**	**0.08**	**−0.08**
q10	U9	0.483*	0.037	−0.018
q11	U10	0.492*	0.011	−0.236*
q12	U11	0.149	0.531*	−0.135
q13	U12	−0.017	0.660*	0.126
q14	U13	0.333*	0.009	0.063
q15	U14	0.155*	0.163*	0.094
q16	U15	−0.008	0.124	0.448*
q17	U16	−0.016	0.536*	0.026
q18	U17	0.425*	−0.185*	−0.007
q20	U18	0.222*	0.568*	−0.019
q22	U19	0.209*	0.02	−0.167*
q23	U20	0.170*	0.247*	−0.118
q25	U21	0.023	0.228*	0.589*
q26	U22	0.537*	0.132	0.07
q27	U23	−0.035	0.257*	0.234*
q28	U24	0.329*	−0.076	−0.435*
q29	U25	0.157*	−0.056	0.008
q30	U26	0.395*	−0.008	0.577*
q31	U27	−0.011	0.557*	−0.146
q32	U28	0.428*	0.164*	−0.012
q33	U29	0.196*	0.296*	−0.137
q34	U30	0.233*	−0.084	−0.147*
q37	U31	0.367*	0.443*	0.029

#### Multidimensional item response theory analysis

Finally, the factor structure of the 30-item instrument was evaluated using a compensatory MIRT model. Compared with the two unidimensional models, the MIRT model demonstrated a substantial improvement in fit, as reflected by markedly lower AIC and BIC values and excellent global fit indices (CFI=0.96, TLI=0.95, RMSEA=0.016 and SRMSR=0.037; table 6).

**Table 6:** Fit indices of the IRT and MIRT models

Model	RMSE	CFI	TLI	AIC	SRMSR	BIC	Alpha	Unidimensional test
IRT (37 items)	0.04	0.627	0.604	24 348.62	0.054	24 670.2	0.572	0.0099
IRT (31 items)	0.04	0.67	0.65	20 320.56	0.056	20 589.99	0.604	0.009
MIRT (30 items)	0.016	0.96	0.95	19 341.29	0.037	19 849.72	0.604	_

IRT, item response theory; MIRT, multidimensional item response theory.

The coefficients of the model, including item slopes (a1–a3) representing discrimination across the three latent factors and item intercepts (d), are presented in online supplemental file 3. Item-level difficulty (MDIFF) and discrimination (MDISC) indices were calculated . Overall, 63% of items showed moderate discrimination, 23% low and 3% very low, while 53% were classified as easy, 17% as medium and 13% as difficult, indicating a balanced and satisfactory spread of item parameters.

**Figure 1:**
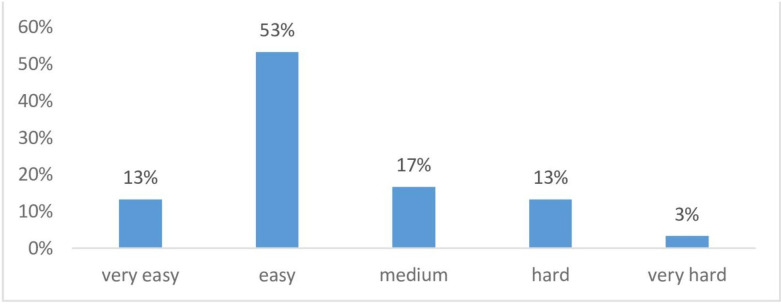
Difficulty of 37 items.

Standardised residual correlations were mostly below 0.20, with only a few reaching 0.30. According to established guidelines, these values suggest minimal local dependence, which is acceptable given the multidimensional nature of the construct and the excellent global model fit. Collectively, these results confirm that the multidimensional MIRT model provides a substantially better and more accurate representation of the data than the unidimensional alternatives. Item-level difficulty (MDIFF) distributions are illustrated in figure 1.

**Figure 2:**
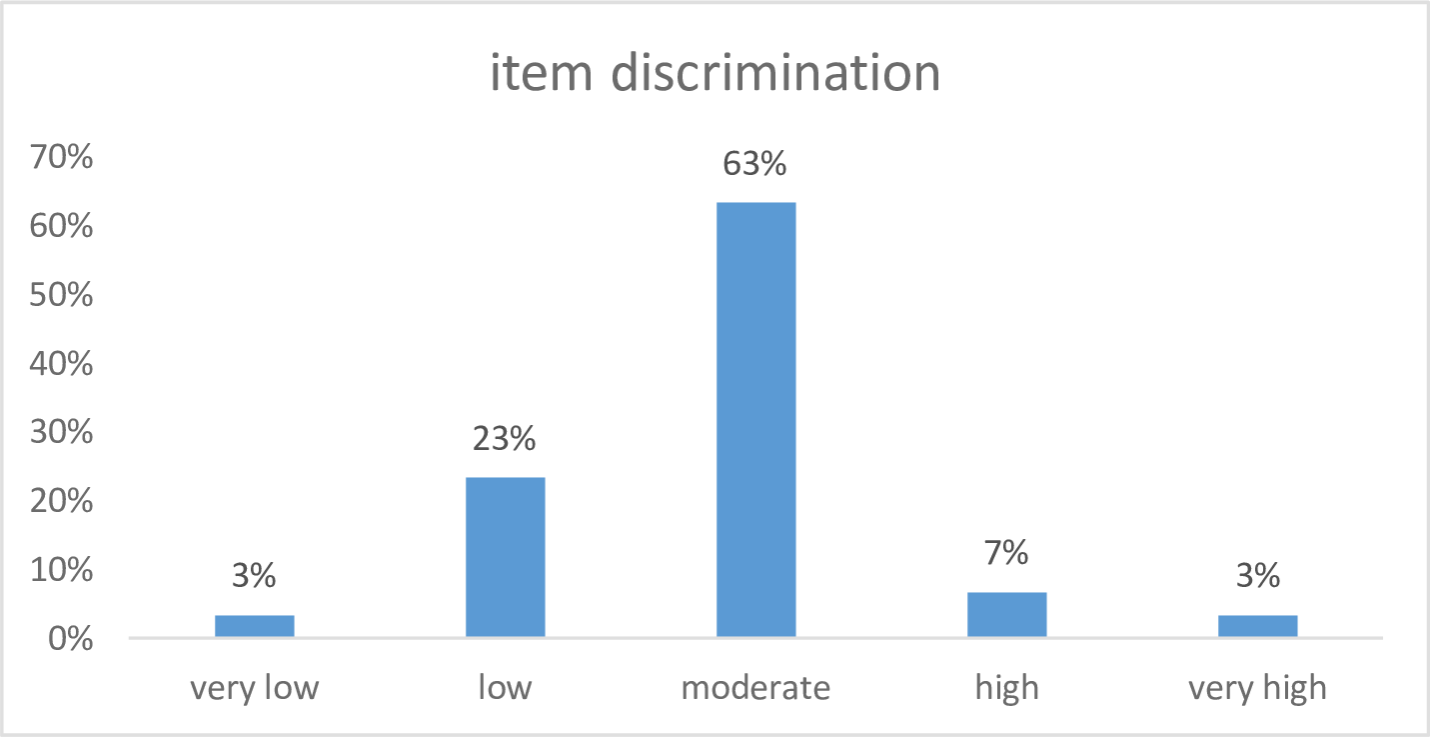
Discrimination of 37 items.

Also, item-level discrimination (MDISC) indices their distributions are illustrated in figure 2

Based on the factor loadings obtained from the three-dimensional IRT approach, each of the 30 items was assigned to one of the three factors (online supplemental file 3). The three extracted factors were confirmed by the researchers. The differential item functioning (DIF) analysis across educational levels revealed no significant results for any item (Δχ² < 3.84, p>0.05), indicating that the scale functioned equivalently among groups. Therefore, although mean total scores differed between groups (t(568) = –3.96, p<0.001, d=0.33), this difference reflects true variations in DHL rather than measurement bias.

### Final questionnaire structure

The final questionnaire was developed with 30 items, which were categorised into three domains based on the content of the questions: risk perception, prevention and preparedness in disasters (14 items); medication-nutritional literacy (11 items); and self-help and emergency literacy (5 items). The scoring system for the questionnaire was a closed format: each correct answer scores 1 point, giving a total score range of 0–30, with higher scores indicating higher DHL in total score and in any of the domains reflecting the proficiency of the patient in that area of literacy. Based on the distribution of scores in our sample, cut-off points were determined using the 33rd and 67th percentiles: *low DHL*: total score ≤18; *medium DHL*: total score 19–21, and *high DHL*: total score ≥22. This approach ensures that the categorisation is data-driven and reflective of the observed distribution in the target population. The Persian and English versions of the DHL questionnaire are included in online supplemental file 4.

## Discussion

The results yielded a three-dimensional structure for the DHL instrument, encompassing disaster risk perception literacy, medication-nutritional literacy and self-help literacy. This refined structure is both empirically and conceptually justified. The integration of medication and nutrition into a single factor reflects their synergistic role in managing diabetes during crises, where dietary and pharmaceutical self-care are inseparable. Similarly, the self-help literacy dimension captures the essential psychological capacities encompassing both self-help and resilience required for proactive crisis response. This structure underscores that effective disaster preparedness for diabetic patients is not a unitary construct but a multifaceted one, integrating general risk awareness with disease-specific management skills and adaptive self-reliance. Consequently, this study successfully developed and validated the first multidimensional instrument to assess DHL specifically for this population, with the final 30-item tool demonstrating robust psychometric properties across three distinct domains. Based on the content coverage of the items within each factor extracted from the MIRT analysis and research team consensus, the final questionnaire was structured into three distinct domains, which were named and defined as follows:

- Disaster eisk literacy (prevention and preparedness domain): This dimension, comprising 14 items, refers to the accurate understanding of potential risks and types of natural disasters (eg, earthquakes and floods) that could affect diabetic patients. It involves a comprehensive grasp of the nature of hazards and severity, their potential impact on health and safety, and the recognition of personal vulnerability. This knowledge empowers patients to take appropriate preventive, preparedness and management actions. The domain covers subcategories such as risk assessment and management, first aid training, self-care and adaptation to group shelter living.
- Medication-nutritional literacy (post-disaster recovery domain): This dimension consists of 11 items focusing on critical post-disaster topics, including blood sugar management, insulin preservation, appropriate nutrition, pre-crisis education and preparedness, and maintaining proper hydration.
- Self-help literacy (focusing on emergency conditions): This dimension includes five items that assess self-care and emergency literacy specific to diabetes management during crises. It addresses the crucial need for immediate and correct responses to complications like hypoglycaemia, stress and dehydration. Awareness and memorisation of emergency contact numbers (eg, for police, fire department and emergency services) are vital components, enabling patients to make appropriate health decisions and ensure faster access to essential healthcare services during disasters.

A key methodological strength was the application of MIRT. While the classical Test Theory provides a foundational approach,^20 26^ our use of MIRT offered superior precision in extracting the underlying factor structure of the instrument. This was critical because health literacy in disasters is an inherently multidimensional construct; a single item often taps into more than one latent ability. The MIRT analysis allowed us to accurately map items onto the three domains, thereby providing a more nuanced and valid representation of the complex DHL ability space than a unidimensional model would permit [24].

Psychometric analyses indicated that most items of the DHLQ exhibited moderate discrimination and were relatively easy, suggesting that the instrument is more sensitive to respondents with low to average ability levels while still covering the latent construct adequately. The MIRT framework provided superior model fit and offered insights into item functioning, highlighting opportunities for future refinement by including more challenging items to better capture higher ability levels. IRT-based DIF analyses showed that item parameters were invariant across educational groups, indicating that the scale functions similarly regardless of education. Observed score differences likely reflect genuine variations in disaster-related health literacy rather than measurement bias. However, further validation in populations with lower literacy is recommended to enhance generalisability. The final scale demonstrated a modest Cronbach’s alpha of 0.61, which is acceptable given the multidimensional nature of the construct and the heterogeneity of DHL responses. Retention of conceptually important items, supported by expert review and IRT analyses, maintained content validity despite moderate internal consistency.

### Practical implications and implementation considerations

The results have direct practical implications for healthcare and emergency management. For healthcare providers, this instrument can serve as a valuable screening and educational needs-assessment tool to identify gaps in preparedness of diabetic patients. It can guide the development of targeted educational programmes focusing on risk perception, medication management and emergency self-care. For emergency management authorities, the domains identified offer a framework for creating patient-centric disaster response protocols and public awareness campaigns. However, successful implementation requires key considerations. Administrators must receive proper training to interpret scores correctly, and it is crucial to emphasise that this tool is designed for preparedness assessment and education, not as a high-stakes diagnostic or triage instrument. Its application should be integrated into broader diabetes management and community preparedness plans.

### Study limitations

While this study provides a significant step forward, several limitations must be acknowledged to qualify the interpretation and generalisability of the findings. The instrument was developed and validated exclusively within an Iranian sample. The cultural context, healthcare system and common disaster types (eg, earthquakes) in Iran may limit the direct applicability of the tool to other settings, such as high-income countries or regions with different disaster profiles (eg, hurricanes). Cross-validation in diverse cultural and socioeconomic populations is essential to establish its broad generalisability. The present study demonstrated strong content, face and construct validity for the DHLQ, as well as robust reliability. However, a key limitation is the lack of criterion and predictive validity testing. Specifically, the relationship between DHLQ scores and actual disaster preparedness behaviours or health outcomes, whether during drills or real events, remains unexamined. Future research should focus on establishing these associations to confirm the practical usefulness of the DHLQ in clinical and public health settings, and ensure that the instrument not only measures DHL theoretically but also reflects real-world preparedness actions. The initial qualitative phases (eg, item generation and expert selection), while conducted rigorously, carry an inherent risk of bias. The perspectives of the selected experts and participants inevitably shaped the content of the final instrument, potentially overlooking other contextual factors influencing DHL. The study used convenience sampling across multiple clinics, which may limit representativeness of the broader diabetic population in Iran. While key demographic factors were analysed, other potential moderators, such as detailed socioeconomic indicators, were not included and warrant exploration in future research.

## Conclusion

This study developed a novel, valid and reliable 30-item multidimensional instrument for assessing DHL in diabetic patients. The three identified domains—disaster risk literacy, medication-nutritional literacy and self-help literacy—provide a comprehensive framework for understanding the critical competencies this vulnerable group needs before, during and after a disaster. The use of MIRT analysis proved advantageous in capturing the complex, multidimensional nature of the construct. In summary, this instrument represents a valuable step toward improving disaster preparedness for diabetic patients. Its usefulness, however, is contingent on an awareness of its developmental limitations, the need for further validation and careful consideration of implementation contexts. We recommend it be used as a foundational tool for assessment and education, within the caveats discussed, to ultimately enhance the safety and health outcomes of this vulnerable population in times of crisis.

### Recommendations for future research

Building on this work, several avenues for future research are proposed. First, there is a need to validate and cross-culturally adapt the instrument in diverse international populations to test its generalisability across different healthcare systems and disaster contexts. Second, future studies should focus on establishing criterion validity by investigating the relationship between DHL scores and objective measures of preparedness or behavioural outcomes during crisis simulations. Finally, longitudinal studies are essential to assess the predictive ability of the tool for long-term preparedness and resilience, and to evaluate the effectiveness of DHL improvement interventions.

### Specific practical applications

The instrument developed in this study has several practical implications. It can serve as: (1) a clinical assessment tool for diabetes educators and physicians to identify specific patient literacy gaps and tailor education; (2) a programme planning and evaluation resource for public health agencies and NGOs to design evidence-based community preparedness initiatives and measure their impact; and (3) a policy-making aid to inform national strategies that protect vulnerable populations in emergencies, positioning DHL as an essential component of community resilience.

## Supplementary material

10.1136/bmjopen-2025-106100 online supplemental file 1

10.1136/bmjopen-2025-106100 online supplemental file 2

10.1136/bmjopen-2025-106100 online supplemental file 3

10.1136/bmjopen-2025-106100 online supplemental file 4

## Data Availability

All data relevant to the study are included in the article or uploaded as supplementary information.
