# Supplementary material for: Design and validation of the Disaster Health Literacy Questionnaire for diabetes patients in Iran: a mixed-methods study
Source: BMJ Open. 2025 Nov 24;15(11):e106100. doi: 10.1136/bmjopen-2025-106100 (PMC12645618; doi:10.1136/bmjopen-2025-106100)
Supplement: online supplemental file 1 [file bmjopen-15-11-s001.pdf]

Supplementary File 1. **CVI, CVR, Item Impact, Kappa, and Cronbach's Alpha for Item Removal**

**Cronbach's alpha**	**Kappa**	**IIC**	**CVR**	**CVI**	**Question**	**Cronbach's alpha**	**Kappa**	**IIC**	**CVR**	**CVI**	**Question**
0.54	0.80*	4.29	1	1	Q20	0.57	0.35*	3.76	1	1	Q1
**0.59**	0.83*	4.29	1	1	Q21	0.56	0.78*	3.93	1	1	Q2
0.57	1.00*	3.15	1	1	Q22	0.57	1.00*	1.85	1	1	Q3
0.56	0.86*	3.70	1	1	Q23	0.57	1.00*	4.29	0/87	1	Q4
**0.58**	0.93*	4.04	1	1	Q24	**0.58**	0.71*	3.64	0/87	1	Q5
0.57	1.00*	3.58	1	1	Q25	0.55	1.00*	3.87	1	1	Q6
0.56	1.00*	4.42	1	1	Q26	0.57	0.84*	4.29	1	1	Q7
0.57	0.92*	3.70	1	1	Q27	0.56	0.84*	4.17	1	1	Q8
0.57	1.00*	4.46	1	1	Q28	0.57	0.80*	4.11	1	1	Q9
0.57	0.86*	3.93	1	1	Q29	0.56	0.78*	4.80	0/6	1	Q10
0.56	1.00*	2.58	0/6	1	Q30	0.56	1.00*	4.73	0/47	1	Q11
0.56	1.00*	2.16	0/87	1	Q31	0.56	0.74*	4.93	1	1	Q12
0.55	0.91*	4.29	0/87	1	Q32	0.56	1.00*	4.04	1	1	Q13
0.56	0.93*	3.70	0/73	1	Q33	0.56	1.00*	4.80	1	1	Q14
0.57	0.93*	2.62	1	1	Q34	0.56	1.00*	4.80	1	1	Q15
**0.58**	1.00*	2.88	1	1	Q35	0.57	1.00*	4.93	1	1	Q16
**0.58**	1.00*	3.15	1	1	Q36	0.56	0.81*	4.87	0/6	1	Q17
0.54	0.88*	2.44	1	1	Q37	0.57	0.93*	4.87	0/73	1	Q18
0.57					Totals	**0.58**	0.93*	3.64	0/73	1	Q19
**p < 0.05**											
