# Supplementary material for: Design and validation of the Disaster Health Literacy Questionnaire for diabetes patients in Iran: a mixed-methods study
Source: BMJ Open. 2025 Nov 24;15(11):e106100. doi: 10.1136/bmjopen-2025-106100 (PMC12645618; doi:10.1136/bmjopen-2025-106100)
Supplement: online supplemental file 2 [file bmjopen-15-11-s002.pdf]

Table 1. Results of Applying the Unidimensional IRT Approach to Each Questionnaire Item

Cronbach's Alpha if Item Deleted	Discrimination Index	Standard Error	Difficulty Index	Response Percentage per Item		Number of Question
				1	0	
0.56	-0.53	0.44	2.4	0.29	0.71	q1
0.56	-0.69	0.69	2.60	0.84	0.16	q2
0.57	-0.43	0.88	2.68	0.75	0.25	q3
0.57	-0.17	2.56	3.79	0.66	0.34	q4
0.58	-0.07	35.65	-18.42	0.23	0.77	q5
0.55	-0.88	0.28	1.23	0.72	0.28	q6
0.57	-0.29	2.96	-5.50	0.17	0.83	q7
0.56	-0.75	0.49	2.00	0.79	0.21	q8
0.58	-0.21	1.03	1.80	0.59	0.41	q9
0.56	-0.67	0.73	2.68	0.84	0.16	q10
0.56	-0.58	0.33	1.17	0.65	0.35	q11
0.56	-0.90	0.32	1.42	0.75	0.25	q12
0.56	-0.92	0.53	2.20	0.85	0.15	q13
0.56	-0.42	0.59	1.81	0.68	0.32	q14
0.56	-0.44	0.26	-0.57	0.44	0.56	q15
0.57	-0.21	1.24	-2.12	0.39	0.61	q16
0.56	-0.70	0.53	2.08	0.79	0.21	q17
0.57	-0.28	0.50	0.93	0.56	0.44	q18
0.58	-0.03	202.65	-43.32	0.24	0.76	q19
0.54	-1.15	0.20	0.88	0.69	0.31	q20
0.59	0.18	3.49	5.12	0.29	0.71	q21
0.57	-0.24	2.55	4.86	0.76	0.24	q22
0.56	-0.54	0.20	-0.44	0.45	0.55	q23
0.59	-0.27	0.35	-0.32	0.48	0.52	q24

Cronbach's Alpha if Item Deleted	Discrimination Index	Standard Error	Difficulty Index	Response Percentage per Item		Number of Question
				1	0	
0.57	-0.44	0.64	1.96	0.70	0.30	q25
0.56	-0.97	0.72	2.73	0.91	0.09	q26
0.57	-0.32	0.35	0.57	0.54	0.46	q27
0.57	-0.16	1.22	1.56	0.56	0.44	q28
0.57	-0.14	2.71	-3.28	0.39	0.61	q29
0.56	-0.58	0.53	1.89	0.74	0.26	q30
0.56	-0.69	0.48	1.91	0.77	0.23	q31
0.55	-0.80	0.36	1.55	0.75	0.25	q32
0.56	-0.60	0.23	0.76	0.61	0.39	q33
0.57	-0.35	0.50	-1.21	0.66	0.34	q34
0.58	-0.17	7.01	8.83	0.82	0.18	q35
0.58	-0.13	4.33	5.09	0.40	0.60	q36
0.54	-1.29	0.23	1.02	0.74	0.26	q37

Table2. Results of Applying the Unidimensional IRT Approach to the Remaining Questionnaire Items

Cronbach's Alpha if Item Deleted	Discrimination Index	Standard Error	Difficulty Index	Response Percentage per Item		Number of Question
				1	0	
0.60	0.84	0.30	3.23	0.29	0.71	q1
0.59	-0.71	0.66	2.52	0.84	0.16	q2
0.60	-0.44	0.87	2.65	0.75	0.25	q3
0.60	-0.20	1.89	3.23	0.66	0.34	q4

Cronbach's Alpha if Item Deleted	Discrimination Index	Standard Error	Difficulty Index	Response Percentage per Item		Number of Question
				1	0	
0.59	-0.84	0.30	1.28	0.72	0.28	q6
0.60	-0.28	3.20	-5.72	0.17	0.83	q7
0.59	-0.78	0.47	1.94	0.79	0.21	q8
0.60	-0.24	0.84	1.60	0.59	0.41	q9
0.59	-0.69	0.71	2.64	0.84	0.16	q10
0.59	-0.58	0.33	1.16	0.65	0.35	q11
0.59	-0.87	0.34	1.46	0.75	0.25	q12
0.59	-0.93	0.53	2.19	0.85	0.15	q13
0.59	-0.45	0.53	1.69	0.68	0.32	q14
0.60	-0.43	0.27	-0.58	0.44	0.56	q15
0.60	-0.26	0.90	-1.78	0.39	0.61	q16
0.59	-0.66	0.57	2.18	0.79	0.21	q17
0.60	-0.30	0.43	0.85	0.56	0.44	q18
0.58	-1.14	0.21	0.89	0.69	0.31	q20
0.60	-0.24	2.74	5.03	0.76	0.24	q22
0.60	-0.50	0.22	-0.46	0.45	0.55	q23
0.60	-0.48	0.56	1.81	0.70	0.30	q25
0.59	-1.00	0.69	2.65	0.91	0.09	q26
0.60	-0.32	0.34	0.56	0.54	0.46	q27
0.61	-0.15	1.31	1.63	0.56	0.44	q28
0.61	-0.13	2.91	-3.39	0.39	0.61	q29
0.59	-0.66	0.45	1.69	0.74	0.26	q30
0.59	-0.65	0.52	2.00	0.77	0.23	q31
0.59	-0.81	0.36	1.53	0.75	0.25	q32
0.59	-0.59	0.24	0.78	0.61	0.39	q33
0.61	-0.14	3.85	4.78	0.66	0.34	q34

Cronbach's Alpha if Item Deleted	Discrimination Index	Standard Error	Difficulty Index	Response Percentage per Item		Number of Question
				1	0	
0.58	-1.25	0.23	1.05	0.74	0.26	q37
