# Supplementary material for: Design and validation of the Disaster Health Literacy Questionnaire for diabetes patients in Iran: a mixed-methods study
Source: BMJ Open. 2025 Nov 24;15(11):e106100. doi: 10.1136/bmjopen-2025-106100 (PMC12645618; doi:10.1136/bmjopen-2025-106100)
Supplement: online supplemental file 3 [file bmjopen-15-11-s003.pdf]

Supplement file 3. Three-dimensional MIRT approach for the remaining 30 questionnaire questions

**Significant Factor loading**			**Cronbach's Alpha if Item Deleted**	**MDIFF**	**MDISC**	**Model coefficients**				**Question**
Factor 3	Factor 2	Factor 1		Difficulty	Discrimination	**d**	**a3**	**a2**	**a1**	
-0.42*			.616	1/1	0/91	-1.04	-0.91	-0.03	0.05	**q1**
	0.37*		.591	-2/2	0/84	1.85	0.32	-0.37	0.68	**q2**
	0.51*		.598	-1/3	0/98	1.32	0.32	-0.83	0.42	**q3**
	0.27*		.604	-1/1	0/64	0.71	0.53	-0.33	0.13	**q4**
		0.39*	.586	-1/2	0/90	1.09	0.04	0.29	0.85	**q6**
		0.23*	.603	2/6	0/67	-1.71	-0.50	-0.30	0.33	**q7**
	0.31*		.590	-1/8	0/85	1.53	0.35	-0.21	0.75	**q8**
	0.43*		.592	-2/1	0/90	1.91	0.28	-0.53	0.67	**q10**
	0.49*		.589	-0/8	1/01	0.76	0.04	-0.77	0.65	**q11**
		0.56*	.588	-1/2	1/19	1.38	-0.56	0.11	1.04	**q12**
		0.57*	.591	-1/8	1/25	2.21	-0.29	0.64	1.03	**q13**
	0.26*		.595	-1/4	0/57	0.78	0.32	-0.24	0.41	**q14**
		0.14*	.597	0/6	0/44	-0.25	0.16	0.03	0.41	**q15**
0.47*			.606	0/6	0/93	-0.53	0.62	0.64	0.27	**q16**
		0.50*	.594	-1/6	0/96	1.54	-0.37	0.45	0.76	**q17**
	0.41*		.599	-0/4	0/80	0.29	0.45	-0.60	0.28	**q18**
		0.59*	.579	-0/8	1/35	1.06	-0.34	0.30	1.27	**q20**
	0.21*		.603	-2/9	0/42	1.22	-0.11	-0.33	0.24	**q22**
		0.25*	.598	0/4	0/57	-0.24	-0.17	-0.11	0.53	**q23**
0.61*			.599	-0/8	1/39	1.13	0.94	0.85	0.57	**q25**
	0.43*		.590	-2/4	1/16	2.78	0.47	-0.40	0.98	**q26**
0.23*			.603	-0/3	0/62	0.19	0.13	0.49	0.36	**q27**
	0.40*		.607	-0/3	0/95	0.30	-0.33	-0.87	0.20	**q28**
	0.16*		.606	1/6	0/28	-0.46	0.17	-0.19	0.12	**q29**
0.68*			.591	-0/9	1/82	1.56	1.58	0.28	0.86	**q30**
		0.56*	.594	-1/3	1/14	1.47	-0.68	0.33	0.85	**q31**
	0.35*		.585	-1/4	0/90	1.27	0.21	-0.32	0.81	**q32**
		0.31*	.591	-0/7	0/66	0.47	-0.22	-0.09	0.62	**q33**
	0.25*		.609	-1/6	0/43	0.71	0.00	-0.41	0.14	**q34**
		0.45*	.575	0/0	1/23	NA	0.00	0.00	1.23	**q37**

*** $p < 0.05$**
