# Supplementary material for: Design and validation of the Disaster Health Literacy Questionnaire for diabetes patients in Iran: a mixed-methods study
Source: BMJ Open. 2025 Nov 24;15(11):e106100. doi: 10.1136/bmjopen-2025-106100 (PMC12645618; doi:10.1136/bmjopen-2025-106100)
Supplement: online supplemental file 4 [file bmjopen-15-11-s004.pdf]

Appendix 1. Disaster Health Literacy Questionnaire Focusing on Diabetic Patients

Dear Participant,

This questionnaire has been developed as part of a Ph.D. dissertation in Medical Library and Information Science titled "Design and Psychometric of a Disaster Health Literacy Questionnaire Focusing on Diabetic Patients: A Mixed-Methods Study" (Ethics Code: IR.MUI.RESEARCH.REC.1400.184 & Registration Code: 3400685, dated December 13, 2021). We kindly ask you to assist us in achieving the study objectives by providing accurate responses to the questions in this questionnaire. Please be assured that the information you provide will be used solely by the researcher for analysis and will remain strictly confidential.

We appreciate your participation

Somayeh Panahi, Ph.D. Medical Library and Information Science, Isfahan University of Medical Sciences

**Demographic Information**	
Gender	Female □ Male □
Age	
University Degree	Higher education□ Below Diploma □ Diploma □
History of Diabetes / Type	Years: … , Type 1 Diabetes □ Type 2Diabetes□
Other Medical Conditions	…………
HbA1C Level (3-month blood glucose)	………….
Height	….cm
Weight	….kg
Experience with Disasters (e.g., flood, earthquake, subsidence, storm, coronavirus, etc.)	**If yes, please specify.**

Questionnaire. Please answer all questions honestly without consulting external resources or assistance

Q	**Disaster Risk Perception Literacy**
1	Where is the best place to store an emergency diabetes kit (containing medication, food, and ID card) before an earthquake occurs?<br>1) Inside a sturdy container near the building exit □<br>2) Inside a box buried in the garden (around the house or neighborhood) □<br>3) In the highest possible location □<br>4) Parking lot □
2	What is the primary and most common cause of land subsidence (silent earthquake)?<br>1) Prolonged rainfall in relatively low-lying urban areas □<br>2) Drought and depletion of underground water resources □<br>3) An increase in land elevation □<br>4) Linear deformation of the land surface □
3	For a person trapped in a vehicle during a flood, which action is the best course of action?<br>1) Slightly open all car windows and exit the vehicle when reaching higher ground □<br>2) If a bridge is present, prefer crossing wooden structures over metal ones □<br>3) Ensure seatbelts are unfastened and car doors are unlocked for easier escape □<br>4) Slightly open one car window and move in the direction of the flood flow for better balance □

4	During a natural disaster, which of the following is correct to prevent potential complications for diabetic foot?<br>1) Applying lotion between the toes □<br>2) Using herbal ointments in case of wounds □<br>3) Wearing open-toe shoes □<br>4) Wearing closed-toe shoes and cotton socks □
5	Why are debris and rubble resulting from disasters around diabetic patients' residences considered a serious threat to them?<br>1) Increased risk of infection and diabetic foot ulcers □<br>2) Psychological and social distress □<br>3) Worsening respiratory problems □<br>4) Blurred vision and retinal bleeding □
6	What is the best action to prevent worsening of the condition, physical injuries, and maintain hygiene and health during disasters?<br>1) Assessing health risks and threats in the disaster-affected area and following official health advisories □<br>2) Given my strong physical resilience, there is no risk of illness for me □<br>3) Due to the severity of post-disaster conditions, diabetic patients are unable to take any specific action □<br>4) Health measures are solely the responsibility of authorities and health experts; individuals have no role □
7	What is the best course of action for a diabetic patient whose relative is trapped under earthquake debris while rescue teams are attempting to help?<br>1) Sitting at the debris removal site, crying, and being restless □<br>2) Taking deep breaths, cooperating with rescue teams as much as possible, and preparing for any news □<br>3) Leaving the debris removal site and isolating oneself while waiting for rescue efforts □<br>4) Ignoring their own condition and exerting maximum effort to find the injured person □
8	After several major earthquakes, what is the most cautious action to take during sheltering?<br>1) Staying calm and praying for divine intervention □<br>2) Keeping identification and insurance documents accessible □<br>3) Ignoring stressful news and sheltering near a corner of the house walls □<br>4) Wearing a whistle and flashlight around the neck □
9	What is the best approach to manage a diabetic foot wound until critical conditions improve and medical access is available?<br>1) Walking to improve blood circulation in the affected foot □<br>2) Cleaning with saline solution or boiled/lukewarm water, drying the wound, and dressing it □<br>3) Keeping the foot moist with a wet dressing □<br>4) Applying herbal oils and traditional remedies to heal the wound □
10	If a lancet is unavailable for blood glucose testing, which of the following should preferably be used as an alternative?<br>Razor blade □<br>Knife □<br>Hypodermic needle □<br>Scissors □
11	What is the most appropriate action to cope with severe anxiety and behavioral disorders following disasters?<br>1) Withdrawing from daily activities and resting in the camp □<br>2) Consulting a psychiatrist or psychologist stationed at the camp □<br>3) Resuming pre-disaster daily routines □<br>4) Ignoring the condition assuming it will improve over time □
12	What is the best action to prevent facing initial challenges caused by natural disasters (floods, earthquakes, subsidence)?

	1) No specific action is needed as natural disasters cannot be predicted or prevented □<br>2) Before potential disasters, identify, evaluate and select safe shelters and emergency supplies (water, food, medicine) in your area □<br>3) Remain in initial location until rescue teams arrive while only preparing basic emergency supplies (water and food) □<br>4) Evacuate immediately upon hearing news from the first available source □
13	For someone living months in group camps without amenities and unable to perform specific activities due to poor conditions, what are the best stress management methods?<br>1) Practicing meditation, talking with friends/family, adapting to limited resources while maintaining available essentials □<br>2) Avoiding social interaction, solitary contemplation about disaster causes and future predictions □<br>3) Attending social gatherings while smoking to overcome insomnia/stress and forget traumatic events □<br>4) Sharing distressing images publicly to solicit food/medical/housing aid from external organizations □
14	When losing loved ones in natural disasters, what is the best approach for personal recovery?<br>1) Taking sedatives/sleeping pills to quickly forget traumatic events □<br>2) Returning to live in the damaged residence regardless of conditions □<br>3) Holding local memorial ceremonies while maintaining social participation □<br>4) Completely suppressing such thoughts even as mental assumptions □
**Medication -Nutritional Literacy**	
15	Mr. Ahmadi, a 33-year-old with type 1 diabetes, accidentally administered too much insulin due to lack of concentration during a natural disaster. Which symptoms is he most likely to experience?<br>1) Flushed face, nausea, and vomiting □<br>2) Fatigue, shortness of breath, and pallor □<br>3) Tremors, anxiety, and sweating □<br>4) Drowsiness and rapid breathing □
16	A diabetic patient with severe kidney pain should reduce consumption of which food group?<br>1) Chicken breast □<br>2) Beef belly □<br>3) Fiber and vegetables □<br>4) Rice and eggs □
17	What complications may rapidly occur if a diabetic patient stops insulin and experiences hyperglycemia during natural disasters?<br>1) Increased body fluids □<br>2) Polydipsia, polyuria, and dry mouth □<br>3) Elevated potassium levels □<br>4) Blurred vision □
18	What is the optimal storage environment for insulin in use during disasters?<br>1) Dry place □<br>2) Freezer compartment □<br>3) Cool place away from light □<br>4) Warm place □
19	Without access to a doctor, when is the best time for a type 1 diabetic to administer regular insulin?<br>1) Only in the mornings □<br>2) 20-30 minutes after meals/simultaneously with meals □<br>3) Only in the evenings □<br>4) 30 minutes before each meal □
20	Which diet is most important for diabetics during disasters?<br>1) Sweets and chocolates □<br>2) Lavash bread □<br>3) Fiber-rich foods, proteins, and whole grain bread □<br>4) Canned foods □

21	What is the best approach regarding herbal medicines and local foods during disasters?<br>1) Consulting family members and relatives □<br>2) Based on personal preference and experience □<br>3) Consulting only with nutritionists and physicians □<br>4) Consulting nutritionists/physicians while considering underlying conditions and personal experience □
22	What are the most common symptoms of diabetic coma in patients at risk of DKA?<br>1) Dry mouth with fruity odor, abdominal pain with vomiting, blood sugar >250 mg/dL □<br>2) Extreme hunger, blood sugar 120-150 mg/dL □<br>3) Fatigue, decreased urination □<br>4) Irritability, blood sugar 60-80 mg/dL □
23	Which statement correctly identifies spoiled or expired insulin?<br>1) Insulin typically spoils 14 days after opening and should be discarded □<br>2) Cloudy insulin that has turned brown □<br>3) Insulin refrigerated at 2-8°C (36-46°F) □<br>4) Insulin stored at room temperature <26°C (79°F) □
24	Which symptoms during disasters indicate hypoglycemia requiring immediate medical attention?<br>1) Dry skin and confusion □<br>2) Decreased heart rate and tremors □<br>3) Burning sensation in lips/tongue □<br>4) Lightheadedness, poor concentration, hunger, sweating, tachycardia □
25	What is the best action for severe insulin syringe shortages during disasters?<br>1) Proper storage and personal reuse of insulin syringe needles □<br>2) Avoiding needle reuse due to infection risks □<br>3) Multiple patients sharing single-use syringes in emergencies □<br>4) Never compromising medical equipment safety and awaiting aid □
**Self-help literacy**	
26	What is the phone number for the Iranian Red Crescent Society?<br>1) 142 □<br>2) 112 □<br>3) 125 □<br>4) 115 □
27	Hossein is a 16-year-old with type 1 diabetes receiving NPH insulin. As a non-medical responder during disasters, when would you anticipate his highest risk of hypoglycemia post-injection?<br>1) 8 hours after injection □<br>2) 4-12 hours after injection □<br>3) 2-3 hours after injection □<br>4) 1 hour after injection □
28	When experiencing palpitations, anxiety, and sweating without glucose monitoring access, what is the most appropriate action?<br>1) Resting in semi-sitting position □<br>2) Taking anti-anxiety medication like propranolol □<br>3) Consuming 4-5 candies or water with sugar cubes □<br>4) Taking half the usual dose of glucose-lowering medication □
29	What physiological challenges might diabetics face during initial disaster hours?<br>1) Hypoglycemia □<br>2) Dehydration, stress and decreased consciousness □<br>3) Seizures □<br>4) Aggression and potential coma □
30	Ms. Rezaei, a 63-year-old diabetic with basic literacy, should immediately report which glucometer reading to emergency responders during disasters?<br>1) "H" symbol (159) on glucometer □<br>2) Blood glucose 120-160 mg/dL □<br>3) Blood glucose >250 mg/dL □<br>4) Blood glucose 70-120 mg/dL □

***Scoring the Questionnaire**

The Disaster Health Literacy Questionnaire, focusing on diabetic patients, is designed in a closed-ended format. For each correct answer, a score of (1) is given, and for an incorrect answer, a score of zero is assigned. Given the number of questions in the questionnaire, the total score for a literate patient in this field is 30. A higher score in each component indicates the patient's ability in that specific dimension of health literacy.

**Answer Key**				
**Number of question**	**1.**	**2.**	**3.**	**4.**
**1)**	*			
**2)**		*		
**3)**			*	
**4)**				*
**5)**	*			
**6)**	*			
**7)**		*		
**8)**				*
**9)**		*		
**10)**			*	
**11)**		*		
**12)**			*	
**13)**	*			
**14)**			*	
**15)**			*	
**16)**		*		
**17)**		*	*	
**18)**			*	
**19)**				*
**20)**			*	
**21)**				*
**22)**	*			
**23)**		*		
**24)**				*
**25)**	*			
**26)**		*		
**27)**	*			
**28)**			*	
**29)**		*		
**30)**	*			
