# Supplementary material for: Design and validation of the Disaster Health Literacy Questionnaire for diabetes patients in Iran: a mixed-methods study
Source: BMJ Open. 2025 Nov 24;15(11):e106100. doi: 10.1136/bmjopen-2025-106100 (PMC12645618; doi:10.1136/bmjopen-2025-106100)
Supplement: Supplementary file 5 [file bmjopen-2025-106100.reviewer_comments.pdf]

PEER REVIEW HISTORY

BMJ Open publishes all reviews undertaken for accepted manuscripts. Reviewers are asked to complete a checklist review form and are provided with free text boxes to elaborate on their assessment. These free text comments are reproduced below.

ARTICLE DETAILS

Title (Provisional)

Design and Validation of the Disaster Health Literacy Questionnaire for Diabetes Patients in Iran: A Mixed-Methods Study

Authors

Panahi, Somayeh; Heidari, Zahra; Heidarpour, Maryam; Atighechian, Golrokh; Ashrafi-rizi, Hasan

---

VERSION 1 - REVIEW

**Reviewer**	**1**
**Name**	**Hany, Mohamed**
**Affiliation**	**Alexandria University**
**Date**	**21-Aug-2025**
**COI**	**None**

This manuscript presents the development and psychometric validation of a novel Disaster Health Literacy Questionnaire (DHLQ) specifically designed for diabetic patients. The study employs a mixed-methods approach, combining qualitative research (scoping review and interviews) with quantitative validation using advanced psychometric methods including Multidimensional Item Response Theory (MIRT). The final instrument comprises 30 items across three dimensions: risk perception literacy, drug-food literacy, and self-help literacy.

The research addresses a significant gap in the literature by creating the first validated disaster health literacy assessment tool specifically tailored for diabetic patients. The methodology is rigorous, employing both classical test theory and modern IRT approaches. The sample size (n=570) is adequate for the psychometric analyses conducted. The study demonstrates strong validity and reliability evidence across multiple domains.

Major Concerns

1. While the study employs sophisticated psychometric methods, several aspects of the methodology require clarification or additional detail. The transition from unidimensional to multidimensional modeling is well-justified, but the specific criteria used for item retention and removal could be more explicitly stated. The authors mention that items with lower

discrimination or higher difficulty were retained “due to the importance of their content,” but this decision-making process lacks clear, pre-specified criteria. This subjective element in item retention could potentially compromise the psychometric integrity of the instrument.

The MIRT analysis, while appropriate for the multidimensional nature of the construct, requires more detailed reporting of model fit indices and comparison with alternative models. The authors should provide more comprehensive fit statistics and discuss the practical implications of the model parameters. Additionally, the assumption testing for MIRT (unidimensionality and local independence) needs more thorough documentation, particularly given that these assumptions were violated in the initial analyses.

2. The study sample, while adequate in size (n=570), is drawn from a specific geographic and cultural context (Iran). The generalizability of the instrument to other populations, healthcare systems, and disaster contexts requires careful consideration. The authors acknowledge this limitation but do not adequately address how cultural, linguistic, and healthcare system differences might affect the instrument’s validity and utility in other settings.

The demographic characteristics of the sample show some concerning patterns that may limit generalizability. The significant differences in scores based on educational level ($p<0.001$) suggest that the instrument may be biased toward individuals with higher educational attainment. This is particularly problematic for a disaster preparedness tool, as vulnerable populations often have lower educational levels and may be most in need of disaster health literacy assessment and intervention.

3. While the study builds on the authors’ previous qualitative work [11, 12], the integration between the theoretical framework derived from the scoping review and the final quantitative instrument could be strengthened. The reduction from five initial dimensions (disaster perception risk literacy, medication literacy, resilience literacy, nutrition literacy, and self-help literacy) to three final dimensions (risk perception literacy, drug-food literacy, and self-help literacy) represents a significant conceptual shift that requires more thorough justification.

The theoretical rationale for combining medication and nutrition literacy into a single dimension needs stronger empirical and conceptual support. These represent distinct domains of knowledge and skill that may have different relationships with disaster preparedness outcomes. The authors should provide more detailed analysis of the factor structure and discuss whether the dimensional reduction enhances or potentially compromises the instrument’s content validity.

Minor Concerns

1. Several statistical reporting issues require attention to enhance the manuscript’s clarity and reproducibility. The Cronbach’s alpha value of 0.57 for the initial 37-item questionnaire is concerning and suggests poor internal consistency. While the authors address this through

item removal, the process and criteria for item deletion need more systematic documentation. The final reliability coefficients should be reported more prominently, and the improvement in reliability following item removal should be quantified.

The presentation of the MIRT results could be enhanced with additional tables showing item parameters, standard errors, and fit statistics. The current presentation focuses primarily on the overall model fit but lacks detailed item-level analysis that would be valuable for future users of the instrument. Additionally, the scoring procedures and interpretation guidelines for the final 30-item instrument are not clearly specified.

2. The convenience sampling method, while practical, introduces potential selection bias that should be more thoroughly acknowledged and discussed. The authors report that 56% of participants were female, but do not provide sufficient detail about other demographic characteristics such as diabetes duration, type of diabetes management, or socioeconomic status. These factors could significantly influence disaster health literacy and should be reported and analyzed as potential moderating variables.

The age range of participants (18-82 years) is broad, but the mean age of 45.57 years suggests a relatively younger sample. Given that older adults with diabetes may face additional challenges during disasters, the instrument's validity for older adults with diabetes requires specific attention. The authors should discuss age-related considerations and potentially recommend age-specific validation studies.

3. While the content validity assessment involved 15 experts from relevant fields, the composition and selection criteria for the expert panel could be better described. The expertise areas (endocrinologists, disaster response specialists, nurses, and medical librarians) are appropriate, but the specific qualifications, experience levels, and potential conflicts of interest are not reported. This information is important for evaluating the credibility of the content validity assessment.

The Content Validity Index (CVI) calculations appear appropriate. Still, the authors should provide more detail about how disagreements among experts were resolved and whether any items were modified based on expert feedback. The perfect S-CVI score of 1.0 seems unusually high and may warrant additional explanation or verification.

4. The manuscript lacks sufficient discussion of practical implementation considerations for the DHLQ. The administration time, required literacy level, and scoring procedures are not clearly specified. For a disaster preparedness tool, ease of administration and interpretation are crucial factors that should be addressed. The authors should provide guidance on who can administer the instrument, how scores should be interpreted, and what cut-off scores might indicate adequate versus inadequate disaster health literacy.

The relationship between DHLQ scores and actual disaster preparedness behaviors or outcomes is not established. While the instrument demonstrates good psychometric properties, its predictive validity for actual disaster response and health outcomes remains

to be demonstrated. This limitation should be acknowledged, and recommendations for future validation studies should be provided.

Language and Style Revision

1. The manuscript title contains a grammatical error that should be corrected. "Design and Validation the Disaster Health Literacy Questionnaire" should be revised to "Design and Validation of the Disaster Health Literacy Questionnaire" (adding "of" before "the"). This error appears consistently throughout the manuscript and should be corrected in all instances.

The abstract effectively summarizes the study but could benefit from more specific reporting of key psychometric indices. The statement "demonstrated strong validity and reliability" should be supported with specific values (e.g., Cronbach's alpha, fit indices). Additionally, the sample size should be mentioned earlier in the abstract methods section for clarity.

2. Several technical terms require clarification or more consistent usage throughout the manuscript. The term "drug-food literacy" in the abstract and results differs from "medication-nutritional literacy" used in the discussion. The authors should establish consistent terminology and define these terms clearly upon first use. The distinction between these concepts and their relationship to traditional health literacy constructs should be explicitly explained.

The abbreviation "DHLQ" is introduced appropriately but should be used consistently throughout the manuscript. Some sections revert to the full term unnecessarily, while others use the abbreviation without sufficient context. A more systematic approach to abbreviation usage would improve readability.

3. The statistical reporting could be enhanced for better clarity and accessibility to readers less familiar with advanced psychometric methods. Terms like "MIRT," "MDISC," and "MDIFF" should be defined more clearly upon first use, with brief explanations of their practical significance. The presentation of statistical results should follow a more consistent format, with appropriate rounding and significance testing clearly indicated.

The reporting of p-values should be more consistent. Some results report exact p-values while others use inequality statements. For transparency and reproducibility, exact p-values should be provided when possible, following current statistical reporting guidelines.

4. Several references require formatting corrections to meet BMJ Open standards. Reference 7 and Reference 9 are incomplete, lacking journal names, volume numbers, and page numbers. Reference 14 includes "DOI." at the end but does not provide the actual DOI. These citations should be completed or corrected as follows:

- Reference 7: Complete journal information needed
- Reference 9: Complete journal information needed

• Reference 14: Either provide the actual DOI (10.1111/j.1744-6570.1975.tb01393.x) or remove "DOI."

• Reference 31: Add missing page numbers

5. The methodology section would benefit from clearer organization and more accessible language. The transition from qualitative to quantitative phases could be better explained, with clearer timelines and participant flow. The description of the MIRT analysis is highly technical and may benefit from a more accessible explanation of why this approach was chosen and what it means for the instrument's validity.

The sample size justification is not clearly provided. While 570 participants is adequate for the analyses conducted, the authors should explain how this sample size was determined and whether it meets recommended guidelines for factor analysis and IRT modeling.

6. The discussion section effectively addresses the study's contributions but could benefit from more balanced consideration of limitations. The authors acknowledge some limitations but may underestimate the impact of cultural specificity and the need for cross-validation in different populations. The practical implications of the findings could be more thoroughly explored, with specific recommendations for implementation in healthcare and emergency management settings.

The conclusion appropriately summarizes the main findings but could be strengthened with more specific recommendations for future research and practical applications. The statement about the instrument serving as a "valuable tool" should be qualified with appropriate caveats about validation needs and implementation considerations.

Novelty Assessment and Literature Comparison

1. Unique Contribution to the Literature

This study makes a significant and novel contribution to the intersection of disaster preparedness and chronic disease management. Through a comprehensive literature review, I identified that this represents the first validated disaster health literacy assessment tool specifically designed for diabetic patients. While several related instruments exist, none combine the disease-specific focus with disaster preparedness in a psychometrically validated format.

The existing literature includes general disaster literacy scales [1], diabetes-specific health literacy tools [2], and disaster preparedness guidelines for chronic diseases [3], but no instrument bridges these domains with the rigor demonstrated in this study. The German Disaster Health Literacy Scale [4] and Turkish Disaster Literacy Scale [5] focus on general populations, while comprehensive diabetes health literacy scales [6] address routine healthcare rather than emergency situations.

2. Comparison with Existing Instruments

General Disaster Health Literacy Tools

The most comparable instruments include the German Disaster Health Literacy Scale (Ewers et al., 2025) [4] and the Turkish Disaster Literacy Scale (Çalışkan & Üner, 2023) [5]. However, these tools differ fundamentally in their target population and scope:

German DIS-HL Scale: This 12-item instrument supplements the HLS19 for general disaster health literacy assessment. While methodologically sound, it lacks disease-specific content and does not address the unique challenges faced by diabetic patients during disasters. The validation approach, while appropriate, is less sophisticated than the MIRT methodology employed in the current study.

Turkish Disaster Literacy Scale: This comprehensive 61-item instrument covers four disaster phases (mitigation, preparedness, response, recovery) for the general population. While thorough, its length may limit practical utility, and it lacks the diabetes-specific focus that makes the current study uniquely valuable.

Diabetes-Specific Health Literacy Instruments

The Comprehensive Diabetes Health Literacy Scale (Lee et al., 2018) [6] represents the gold standard for diabetes health literacy assessment in routine healthcare settings. This 14-item instrument measures informational, numeracy, and communicative health literacy with strong psychometric properties (Cronbach's $\alpha = 0.91$). However, it focuses entirely on routine diabetes management and lacks any disaster-specific content.

The current study's innovation lies in adapting health literacy assessment to emergency contexts while maintaining diabetes-specific relevance. The three-dimensional structure (risk perception literacy, medication-nutritional literacy, self-help literacy) represents a novel conceptualization that addresses the unique intersection of disaster preparedness and diabetes management.

3. Methodological Innovation

The use of Multidimensional Item Response Theory (MIRT) for disaster health literacy assessment represents a methodological advancement over existing instruments. Most comparable tools rely on Classical Test Theory approaches, which may not adequately capture the complexity of multidimensional constructs. The MIRT approach allows for more nuanced understanding of item functioning and provides superior measurement precision across different ability levels.

The mixed-methods development approach, beginning with qualitative research [11, 12] and progressing to sophisticated quantitative validation, demonstrates methodological rigor that exceeds most existing instruments in this domain. The integration of scoping review findings with interview data provides a strong theoretical foundation that is often lacking in instrument development studies.

4. Practical Significance and Gap Filling

This instrument addresses a critical gap identified in disaster preparedness literature. Diabetic patients represent a particularly vulnerable population during disasters due to their dependence on medications, dietary requirements, and potential complications [7, 8]. Existing disaster preparedness resources for diabetic patients consist primarily of educational materials and checklists rather than validated assessment tools [9, 10].

The DHLQ enables systematic assessment of disaster preparedness knowledge and skills specific to diabetes management, facilitating targeted interventions and program evaluation. This capability is essential for emergency management agencies, healthcare systems, and public health organizations working to improve disaster resilience in vulnerable populations.

5. Limitations in Novelty Claims

While the study makes important contributions, some limitations in novelty should be acknowledged. The conceptual framework builds heavily on existing health literacy and disaster preparedness theories, and the dimensional structure shows similarities to established health literacy models. The innovation lies primarily in the specific application to diabetic patients in disaster contexts rather than fundamental theoretical advancement.

Additionally, while the instrument is novel in its specific focus, the broader concept of disaster health literacy has been explored in various forms. The authors appropriately acknowledge their previous work [11, 12] as foundational to this study, which demonstrates appropriate scholarly progression rather than entirely novel concept development.

6. Future Research Implications

This study opens several important avenues for future research, including cross-cultural validation, predictive validity studies, and intervention effectiveness evaluation. The instrument provides a foundation for developing similar tools for other chronic conditions and for studying the relationship between disaster health literacy and actual emergency outcomes.

The methodological approach demonstrated here could serve as a model for developing disaster-specific health literacy instruments for other vulnerable populations, potentially leading to a family of related assessment tools that address various chronic conditions and special needs populations.

References used:

[1] Çalışkan, C., & Üner, S. (2023). Measurement of disaster literacy in Turkish society: Disaster Literacy Scale (DLS) design and development process. Disaster Medicine and Public Health Preparedness, 17, e211.

[2] Lee, E. H., Lee, Y. W., Lee, K. W., Nam, M., & Kim, S. H. (2018). A new comprehensive diabetes health literacy scale: Development and psychometric evaluation. International Journal of Nursing Studies, 88, 1-8.

[3] Tomio, J., & Sato, H. (2014). Emergency and disaster preparedness for chronically ill patients: a review of recommendations. Open Access Emergency Medicine, 6, 69-79.

[4] Ewers, M., Beckert, J., Griese, L., et al. (2025). Disaster Health Literacy – development and validation of a short measurement instrument in German to supplement the HLS19 instruments. Frontiers in Public Health, 13, 1589705.

[5] Çalışkan, C., & Üner, S. (2022). Measurement of disaster literacy in Turkish society: Disaster Literacy Scale (DLS) design and development process. Disaster Medicine and Public Health Preparedness, 17, e211.

[6] Lee, E. H., Lee, Y. W., Lee, K. W., Nam, M., & Kim, S. H. (2018). A new comprehensive diabetes health literacy scale: Development and psychometric evaluation. International Journal of Nursing Studies, 88, 1-8.

[7] Jenkins, A. J., Ogle, G. D., Scibilia, R., et al. (2024). People who need insulin are particularly vulnerable in disasters and conflicts. BMJ, 385, q1109.

[8] Allweiss, P. (2019). Diabetes and disasters: recent studies and resources for preparedness. Current Diabetes Reports, 19, 131.

[9] American Diabetes Association. (2018). Diabetes Disaster Preparedness Plan. Available at: https://diabetes.org/sites/default/files/2023-10/DDRCPatientPreparednessPlan2018Short.pdf

[10] Centers for Disease Control and Prevention. (2024). Diabetes Care During Emergencies. Available at: https://www.cdc.gov/diabetes/articles/diabetes-care-emergencies.html

[11] Panahi, S., Atighechian, G., Heidarpour, M., et al. (2024). Disaster health literacy for diabetics: A scoping review towards a framework. Journal of Education and Health Promotion, 13(1), 475.

[12] Panahi, S., Heidarpour, M., Atighechian, G., et al. (2023). Improving Diabetes Self-Help in Natural Disasters: Qualitative Parameters and Recommendations. Iranian Red Crescent Medical Journal, 25(9), e3059.

**Reviewer**	**2**
**Name**	**Peimani , Maryam**
**Affiliation**	**Tehran University of Medical Sciences**
**Date**	**07-Sep-2025**
**COI**	**None**

Abstract

1. The phrase "content validity (CVI & CVI)" in the Methods section is a clear typo, likely intended as "CVI & CVR" (Content Validity Index and Content Validity Ratio), as detailed in the full Methods section.

2. In the Results section, “strong validity and reliability” is vague; please specify key metrics (e.g., Cronbach's alpha, acceptable fit indices) to show the validity and reliability obtained.

Introduction

The Introduction effectively sets the stage by outlining the global burden of diabetes, linking it to health literacy, highlighting vulnerabilities in disaster contexts, identifying a clear research gap (lack of a validated tool for disaster health literacy in diabetic patients), and stating the study's aim. However, I have some minor comments to strengthen this section:

1. The definition of "disaster health literacy" is embedded ("encompasses the skills and knowledge needed to effectively mitigate risks and respond to emergencies"), but I think it could be more explicitly defined or referenced earlier. Please consider adding a brief citation to a standard definition (e.g., from WHO or existing literature) to ground it theoretically, especially since this is central to the study.

2. There are a few run-on sentences and missing punctuation that could be smoothed for better flow. For example:

"Furthermore, volunteer organizations often lack specialized resources for diabetic patients and strategies for maintaining proper medication storage conditions post-disaster Disaster health literacy encompasses...".

This appears to be a run-on; insert a dot or semicolon: "...post-disaster. Disaster health literacy encompasses..."

3. While the global burden is covered well, the section could acknowledge regional variations (e.g., higher disaster risks in low- and low-middle-income countries like Iran, where the study is based) to broaden its appeal. Additionally, the claim that "two-thirds of diabetic individuals lack adequate knowledge about disaster preparedness" (ref 7) is strong—ensure this reference is robust and generalizable, as it seems to be context-specific.

Methods

1. This section mentions sample sizes (e.g., 15 experts for content validity, 15 patients for face validity, 30 for test-retest, 570 for construct validity) but provides no information on how participants were selected, recruited, or sampled (e.g., convenience, purposive, random?). Inclusion/exclusion criteria (e.g., type 1 vs. type 2 diabetes, duration of illness, literacy levels) are absent, as is the study setting (e.g., specific clinics or regions in Iran). Demographics (e.g., age, gender, education) are not described, which is critical for assessing generalizability, especially in a culturally specific context like disaster preparedness. I recommend adding a subsection on "Participants and Sampling" with these details to enhance reproducibility.

2. While 570 for construct validity is reasonable for IRT/MIRT, there is no rationale or power calculation provided.

3. Minor errors persist, e.g., "2 of medical librarians" should be "2 medical librarians"; "Ave/CVI-S" could be spelled out as "Scale-level Content Validity Index (S-CVI/Ave)" on first use.

Findings/Results

1. Use consistent terms (e.g., "Findings" vs. "Results" in abstract)

2. The Results flow logically but I suggest using subheadings (e.g., "Item Reduction," "Reliability," "Construct Validity") for better navigation.

Discussion

1. I think the detailed comparison of IRT/MIRT vs. CTT (with refs 20-30) is informative but dominates the opening, reading more like a methods extension than interpretation. I suggest condensing this to focus on how MIRT enhanced your specific findings (e.g., precise domain extraction) and integrating it with results interpretation.

2. While limitations are explicitly stated (e.g., MIRT assumptions, retention of low-discrimination items, focus on self-help potentially overlooking contextual factors), they are somewhat understated and could be expanded for transparency. For instance, discuss how the Iranian sample limits generalizability to other cultural/disaster contexts (e.g., high-income vs. low-resource settings). Also, address potential biases in qualitative phases (e.g., expert selection) or the lack of criterion validity testing (e.g., against existing health literacy tools).

VERSION 1 - AUTHOR RESPONSE

Reviewer 1:

Thank you for the opportunity to improve our manuscript, we hope these revisions meet with your approval.

N	Reviewer 1' Comments to Author	Author (s)' Response
	Major Concerns<br><br>1. While the study employs sophisticated psychometric methods, several aspects of the methodology require clarification or additional detail. The transition from unidimensional to multidimensional modeling is well-justified, but the specific criteria used for item retention and removal could be more explicitly stated. The authors mention that items with lower discrimination or higher difficulty were retained "due to the importance of their content," but this	We thank the reviewer for this constructive comment and appreciate the opportunity to clarify this point. In our multidimensional IRT (MIRT) analysis, all items met acceptable psychometric thresholds.<br>As illustrated in Figures 1 and 2, discrimination and difficulty distributions indicate an overall balanced and satisfactory spread of item parameters. Therefore, the previous statement suggesting that certain items were retained "due to the importance of their content" was inaccurate

N	Reviewer 1' Comments to Author	Author (s)' Response
	decision-making process lacks clear, pre-specified criteria. This subjective element in item retention could potentially compromise the psychometric integrity of the instrument.<br>The MIRT analysis, while appropriate for the multidimensional nature of the construct, requires more detailed reporting of model fit indices and comparison with alternative models. The authors should provide more comprehensive fit statistics and discuss the practical implications of the model parameters. Additionally, the assumption testing for MIRT (unidimensionality and local independence) needs more thorough documentation, particularly given that these assumptions were violated in the initial analyses.	and has now been removed from the Discussion section. We confirm that all item retention decisions were based solely on psychometric evidence derived from the MIRT analysis.<br>Regarding assumptions testing:<br>a. The results of unidimensionality assumption using a modified parallel analysis was added in the manuscript (see paragraph 2 of section A: Item Response Theory (IRT) Analysis and table 4). These results were significant across both the initial (37-item) and reduced (31-item) IRT models, confirming that the data did not conform to a strictly unidimensional structure.<br>b. The results of the local independence analysis were also added to the manuscript (see paragraph 3 of section A: Item Response Theory (IRT) Analysis and table4), revealing the presence of local dependence in both the IRT and MIRT models, indicating that the assumption of local independence was violated in both unidimensional IRT models.<br>c. We also added the results of Q3 residual analysis within the MIRT framework to further examine local dependence (see paragraph 3 of section C: Multidimensional Item Response Theory (MIRT) Analysis) The results showed that most standardized residual correlations were below 0.20, and only a few item pairs reached up to 0.30. According to established guidelines, these values indicate minimal local dependence, which is acceptable given the multidimensional structure of the instrument and the excellent overall model fit.<br>We have revised the manuscript to include a more detailed presentation of the MIRT model fit indices and comparisons with alternative IRT models: Specifically, the MIRT (30-item) model demonstrated excellent fit (CFI = 0.96, TLI = 0.95, RMSEA = 0.016, SRMSR = 0.037, AIC = 19341.29, BIC = 19849.72) (table 4) compared to the unidimensional IRT models (CFI = 0.627–0.67, TLI = 0.604–0.65) (table 4). These results confirm that a multidimensional model provides a substantially better representation of the data.
	The study sample, while adequate in size (n=570), is drawn from a specific geographic and cultural context (Iran). The generalizability of	We appreciate the reviewer's comment regarding the generalizability of our instrument. As with any newly developed

N	Reviewer 1' Comments to Author	Author (s)' Response
	the instrument to other populations, healthcare systems, and disaster contexts requires careful consideration. The authors acknowledge this limitation but do not adequately address how cultural, linguistic, and healthcare system differences might affect the instrument's validity and utility in other settings.<br><br>The demographic characteristics of the sample show some concerning patterns that may limit generalizability. The significant differences in scores based on educational level (p<0.001) suggest that the instrument may be biased toward individuals with higher educational attainment. This is particularly problematic for a disaster preparedness tool, as vulnerable populations often have lower educational levels and may be most in need of disaster health literacy assessment and intervention.	tool, the current questionnaire was validated within the Iranian context, and its application in other cultural or healthcare settings would require separate psychometric evaluation.<br>Thank you for this valuable comment. We agree that educational level can influence disaster-related health literacy and thus examined whether the instrument functions equivalently across education groups. Differential item functioning (DIF) across educational levels was examined using the graded response model in R (mirt package). Model comparisons showed no significant DIF for any item ($\Delta\chi^2 < 3.84$, $p > 0.05$), indicating that item parameters were invariant across groups. Therefore, the observed score difference between education levels likely reflects genuine variations in disaster health literacy rather than measurement bias.We have clarified these points in the Results (see last paragraph of section C: Multidimensional Item Response Theory (MIRT) Analysis) and Discussion sections (paragraph 3).
	While the study builds on the authors' previous qualitative work [11, 12], the integration between the theoretical framework derived from the scoping review and the final quantitative instrument could be strengthened. The reduction from five initial dimensions (disaster perception risk literacy, medication literacy, resilience literacy, nutrition literacy, and self-help literacy) to three final dimensions (risk perception literacy, drug-food literacy, and self-help literacy) represents a significant conceptual shift that requires more thorough justification.<br>The theoretical rationale for combining medication and nutrition literacy into a single dimension needs stronger empirical and conceptual support. These represent distinct domains of knowledge and skill that may have different relationships with disaster preparedness outcomes. The authors should provide more detailed analysis of the factor structure and discuss whether the dimensional reduction enhances or potentially compromises the instrument's content validity.	We appreciate the reviewer's insightful comment. We agree that theoretical coherence is important; however, it is not uncommon for empirical analyses to yield a factor structure that differs from the initially hypothesized model. In the present study, we systematically explored models ranging from two to six factors using exploratory factor analysis (EFA) repeated five times.<br>After careful examination by the research team, the three-factor model demonstrated the most interpretable structure and the best statistical performance, supported by the IRT/MIRT fit indices.<br>We thank the reviewer for this important comment. The reduction from five to three dimensions therefore reflects an evidence-based refinement rather than a loss of conceptual integrity. Importantly, the retained factors still capture the core domains of disaster health literacy — namely, risk perception, drug–food management, and self-help literacy. We have clarified this rationale in the revised manuscript (see paragraph one of section B: Exploratory Factor Analysis).<br>That is correct that medication and nutrition literacy are distinct domains. However, in the context of disaster preparedness for

N	Reviewer 1' Comments to Author	Author (s)' Response
		chronic diseases, our empirical analysis revealed that these two skill sets are highly correlated and loaded onto a single factor. This suggests that in a disaster scenario, the knowledge and skills for managing medication and nutrition are functionally intertwined for patients. Therefore, combining them into a single 'drug-food management' dimension enhances the instrument's practical relevance and parsimony without compromising its content validity. This dimensional reduction is not only empirically supported by the factor analysis but is also theoretically justified. (see Discussion)
	Minor Concerns<br>1. Several statistical reporting issues require attention to enhance the manuscript's clarity and reproducibility. The Cronbach's alpha value of 0.57 for the initial 37-item questionnaire is concerning and suggests poor internal consistency. While the authors address this through item removal, the process and criteria for item deletion need more systematic documentation. The final reliability coefficients should be reported more prominently, and the improvement in reliability following item removal should be quantified.<br><br>The presentation of the MIRT results could be enhanced with additional tables showing item parameters, standard errors, and fit statistics. The current presentation focuses primarily on the overall model fit but lacks detailed item-level analysis that would be valuable for future users of the instrument. Additionally, the scoring procedures and interpretation guidelines for the final 30-item instrument are not clearly specified.	We appreciate the reviewer's valuable comments. The item reduction process and reliability improvement are now described in more detail. The results of the initial and reduced IRT analyses are presented in Tables 1 and 2 of supplement 1, which include item difficulty, discrimination indices, and Cronbach's alpha values after each item deletion. Items were removed based on low item–total correlation, improvement in alpha, and inappropriate IRT parameters, supported by expert judgment. In total, six items were removed during the IRT phase and one during exploratory factor analysis, increasing Cronbach's alpha from 0.57 to 0.61. Detailed MIRT item parameters, standard errors, and fit indices are fully reported in Table 6, and a clearer reference to this table has been added to the Results section and paragraph 3 of Discussion sections.<br>Although the final Cronbach's alpha of 0.61 is modest, it is considered acceptable given the exploratory nature of this study and the multidimensional construct being measured. The scale covers diverse aspects of Disaster Health Literacy where heterogeneity in responses is expected, which naturally limits internal consistency. Moreover, the focus on retaining conceptually important items, supported by expert judgment and IRT analyses, ensures that the scale remains content-valid and interpretable despite the moderate alpha (Discussion sections (paragraph 4)<br>Thank you for your comment. The scoring procedure for the final 30-item Disaster Health Literacy Questionnaire is

N	Reviewer 1' Comments to Author	Author (s)' Response
		straightforward and added to the manuscript (section Final Questionnaire Structure, paragraph1)
	2. The convenience sampling method, while practical, introduces potential selection bias that should be more thoroughly acknowledged and discussed. The authors report that 56% of participants were female, but do not provide sufficient detail about other demographic characteristics such as diabetes duration, type of diabetes management, or socioeconomic status. These factors could significantly influence disaster health literacy and should be reported and analyzed as potential moderating variables.<br><br>The age range of participants (18-82 years) is broad, but the mean age of 45.57 years suggests a relatively younger sample. Given that older adults with diabetes may face additional challenges during disasters, the instrument's validity for older adults with diabetes requires specific attention. The authors should discuss age-related considerations and potentially recommend age-specific validation studies.	We appreciate the reviewer's constructive comment. In response, we have expanded the demographic description of participants to include additional variables such as diabetes duration, type of diabetes, comorbidities, obesity status, education level, and prior hazard experience (see paragraph one of Demographic statistics of participants' section, Table 3). Furthermore, we have acknowledged the potential selection bias related to the convenience sampling method in the Limitations section. (paragraph 4 of limitations)<br>We also examined the correlations between age and diabetes duration with total disaster health literacy scores, which were not statistically significant ($p > 0.05$) (see paragraph tow of Construct Validity part) (Demographic statistics of participants section, paragraph2).
	3. While the content validity assessment involved 15 experts from relevant fields, the composition and selection criteria for the expert panel could be better described. The expertise areas (endocrinologists, disaster response specialists, nurses, and medical librarians) are appropriate, but the specific qualifications, experience levels, and potential conflicts of interest are not reported. This information is important for evaluating the credibility of the content validity assessment.<br><br>The Content Validity Index (CVI) calculations appear appropriate. Still, the authors should provide more detail about how disagreements among experts were resolved and whether any items were modified based on expert feedback. The perfect S-CVI score of 1.0 seems unusually high and may warrant additional explanation or verification.	Thanks for the comment. Table 1 fully shows the demographic characteristics of each stage.<br>We thank the reviewer for this comment. The Content Validity Index (CVI) was calculated following established guidelines. When disagreements among experts arose, they were resolved through discussion and consensus within the expert panel. Several items were modified slightly based on their feedback to improve clarity and relevance (see Content Validity Index of methods section)<br>Regarding the perfect S-CVI score of 1.0, this reflects that all items were rated as relevant by all experts in our panel. While this is an unusually high score, it is plausible given the careful item development process and the selection of knowledgeable experts. We have double-checked the calculations to ensure accuracy.
	4. The manuscript lacks sufficient discussion of practical implementation considerations for the DHLQ. The administration time, required literacy level, and scoring procedures are not clearly specified. For a disaster preparedness	Thank you for your comment. In the discussion and conclusion section, revisions were made, and executive considerations were also provided.

N	Reviewer 1' Comments to Author	Author (s)' Response
	tool, ease of administration and interpretation are crucial factors that should be addressed. The authors should provide guidance on who can administer the instrument, how scores should be interpreted, and what cut-off scores might indicate adequate versus inadequate disaster health literacy.<br>The relationship between DHLQ scores and actual disaster preparedness behaviors or outcomes is not established. While the instrument demonstrates good psychometric properties, its predictive validity for actual disaster response and health outcomes remains to be demonstrated. This limitation should be acknowledged, and recommendations for future validation studies should be provided.	The scoring procedure for the final 30-item Disaster Health Literacy Questionnaire added to the manuscript (section Final Questionnaire Structure, paragraph1).<br>We appreciate the reviewer's comment regarding the predictive validity of the DHLQ. As noted, while the instrument demonstrated strong reliability and construct validity, its ability to predict actual disaster preparedness behaviors or health outcomes could not be assessed in the present study. This was due to the lack of comparable instruments or practical measures for evaluating real-world disaster responses among diabetes patients in Iran. We have acknowledged this as a limitation in the Discussion section and suggested that future research should explore the relationship between DHLQ scores and actual preparedness actions or disaster-related health outcomes to establish its predictive validity and further enhance the instrument's practical utility. (paragraph 3 of limitation revised)
	Language and Style Revision<br>1. The manuscript title contains a grammatical error that should be corrected. "Design and Validation the Disaster Health Literacy Questionnaire" should be revised to "Design and Validation of the Disaster Health Literacy Questionnaire" (adding "of" before "the"). This error appears consistently throughout the manuscript and should be corrected in all instances.<br><br>The abstract effectively summarizes the study but could benefit from more specific reporting of key psychometric indices. The statement "demonstrated strong validity and reliability" should be supported with specific values (e.g., Cronbach's alpha, fit indices). Additionally, the sample size should be mentioned earlier in the abstract methods section for clarity.	The grammatical error in the title and throughout the manuscript has been corrected to "Design and Validation **of** the Disaster Health Literacy Questionnaire...".<br><br>2. The abstract has been revised to include the specific sample size (N=570) in the methods section and now reports key psychometric indices (e.g., Cronbach's α, RMSEA, CFI) to support the validity and reliability claims.
	2. Several technical terms require clarification or more consistent usage throughout the manuscript. The term "drug-food literacy" in the abstract and results differs from "medication-nutritional literacy" used in the discussion. The authors should establish consistent terminology and define these terms clearly upon first use. The distinction between these concepts and their relationship to traditional health literacy constructs should be explicitly explained.	Thank you for this precise comment. The term "drug-food literacy" has been standardized to "medication-nutritional literacy" throughout the manuscript, with a clear definition provided upon its first use. Its relationship to traditional health literacy constructs has been explicitly explained in the discussion section.<br>We thank you for this pertinent remark. As per your guidance, the usage of the

N	Reviewer 1' Comments to Author	Author (s)' Response
	The abbreviation "DHLQ" is introduced appropriately but should be used consistently throughout the manuscript. Some sections revert to the full term unnecessarily, while others use the abbreviation without sufficient context. A more systematic approach to abbreviation usage would improve readability.	abbreviation "DHLQ" has been systematically reviewed throughout the manuscript. We have ensured it is used consistently and appropriately after its initial definition, avoiding unnecessary reversion to the full term.
	3. The statistical reporting could be enhanced for better clarity and accessibility to readers less familiar with advanced psychometric methods. Terms like "MIRT," "MDISC," and "MDIFF" should be defined more clearly upon first use, with brief explanations of their practical significance. The presentation of statistical results should follow a more consistent format, with appropriate rounding and significance testing clearly indicated.<br><br>The reporting of p-values should be more consistent. Some results report exact p-values while others use inequality statements. For transparency and reproducibility, exact p-values should be provided when possible, following current statistical reporting guidelines.	We sincerely thank the reviewer for this valuable feedback.<br><br>Definition of Technical Terms: As suggested, the specialized terms "MIRT" (Multidimensional Item Response Theory), "MDISC" (Multidimensional Discrimination), and "MDIFF" (Multidimensional Difficulty) are now clearly defined upon their first use in the dedicated MIRT section. We have included brief explanations of their practical significance to make the methodology accessible to a broader audience.<br><br>The reporting of p-values has been unified.
	4. Several references require formatting corrections to meet BMJ Open standards. Reference 7 and Reference 9 are incomplete, lacking journal names, volume numbers, and page numbers. Reference 14 includes "DOI." at the end but does not provide the actual DOI. These citations should be completed or corrected as follows:<br><br>• Reference 7: Complete journal information needed<br>• Reference 9: Complete journal information needed<br>• Reference 14: Either provide the actual DOI (10.1111/j.1744-6570.1975.tb01393.x) or remove "DOI."<br>• Reference 31: Add missing page numbers	Thank you very much for your careful review and valuable comments. We have carefully revised the references as suggested. The formatting issues for References 7, 9, 14, and 31 have been corrected to fully comply with the BMJ Open standards. The changes are as follows:<br><br>Reference 7 & 9: The complete journal information (journal name, volume, and page numbers) has been added.<br>(R7: Ghazanchaei e, Allahbakhshi K, Khorasani-Zavareh D, et al. Natural Disasters Preparedness Knowledge of Patients with Chronic Diseases in Iran: A Descriptive Study on Patients with Diabetes and Chronic Obstructive Pulmonary Disease in Masih Daneshvari Hospital, Tehran, Iran. Iranian Red Crescent Medical Journal (IRCMJ) 2022;24(4):-. doi: 10.32592/ircmj.2022.24.4.1688<br>R9: Karahan E Eİ, Yönem A. The Effects of the COVID-19 Pandemic Process on the Treatment and Follow-up of Diabetes Mellitus in Type 2 Diabetic Patients. Hamidiye Med J. 2024 Mar 19;5(1):13-20. doi: 10.4274/hamidiyemedj.galenos.2024.63835.

N	Reviewer 1' Comments to Author	Author (s)' Response
		R14: The correct DOI (doi: 10.1111/j.1744-6570.1975.tb01393.x) has been provided.<br>R31: The missing page numbers have been added.(30(3):439-48.
	5. The methodology section would benefit from clearer organization and more accessible language. The transition from qualitative to quantitative phases could be better explained, with clearer timelines and participant flow. The description of the MIRT analysis is highly technical and may benefit from a more accessible explanation of why this approach was chosen and what it means for the instrument's validity. The sample size justification is not clearly provided. While 570 participants is adequate for the analyses conducted, the authors should explain how this sample size was determined and whether it meets recommended guidelines for factor analysis and IRT modeling.	We sincerely appreciate the reviewer's helpful and constructive comments. In response, several revisions were made to improve the clarity, structure, and accessibility of the Methods section:<br><br>• Structural organization: The section was reformatted using clear subheadings, bullet points, and logical sequencing to make the methodology easier to follow.<br><br>• Transition between phases: A short sentence and a schematic description were added to clarify the transition from the qualitative to the quantitative phase, outlining how the qualitative results informed item development and subsequent psychometric testing.<br><br>• Reduction of technical details: Overly technical and redundant statistical explanations were reduced to make the text more concise and accessible to a broader audience, while maintaining methodological rigor.<br><br>• Clarification of MIRT rationale: Additional sentences were added to briefly explain the rationale for selecting the Multidimensional Item Response Theory (MIRT) approach and its advantages. Specifically, it was noted that MIRT allows for the simultaneous estimation of multiple latent traits, which better represents the multidimensional nature of the DHLQ and strengthens evidence for construct validity.<br><br>These changes have improved the organization, flow, and interpretability of the methodology section while addressing the reviewer's valuable recommendations.<br><br>Thank you for your comment. The sample size of 570 participants was determined based on commonly recommended guidelines for both factor analysis and IRT modeling. For exploratory and confirmatory factor analysis, a sample of at least 5–10 participants per item is generally suggested; with the initial 37-item questionnaire, our

N	Reviewer 1' Comments to Author	Author (s)' Response
		sample meets this criterion (37 × 10 = 370). For IRT and MIRT analyses, larger samples improve parameter estimation stability, and previous studies suggest that 500–600 participants are sufficient for dichotomous item calibration in instruments of similar length. After item reduction, the final 30-item instrument still maintains an adequate subject-to-item ratio, ensuring robust estimation of item parameters and model fit (see participants part of method section)
	6. The discussion section effectively addresses the study's contributions but could benefit from more balanced consideration of limitations. The authors acknowledge some limitations but may underestimate the impact of cultural specificity and the need for cross-validation in different populations. The practical implications of the findings could be more thoroughly explored, with specific recommendations for implementation in healthcare and emergency management settings.<br>The conclusion appropriately summarizes the main findings but could be strengthened with more specific recommendations for future research and practical applications. The statement about the instrument serving as a "valuable tool" should be qualified with appropriate caveats about validation needs and implementation considerations.	We sincerely thank you for their insightful and constructive comments, which have significantly helped us improve the quality of our manuscript. We have carefully addressed each point raised.<br>•Point 1 (Balanced Limitations & Practical Implications): We agree with the reviewer that a more balanced consideration of limitations and a deeper exploration of practical implications would strengthen the discussion. Accordingly, we have substantially expanded the 'Study Limitations' subsection within the Discussion section. We now provide a detailed and candid discussion of the impact of cultural specificity, the necessity for cross-validation in different populations, and other key limitations. Furthermore, we have added a new dedicated subsection titled 'Practical Implications and Implementation Considerations' under the Discussion. This section explicitly outlines specific recommendations for implementing our findings in healthcare and emergency management settings, directly addressing the reviewer's valuable suggestion.<br>•Point 2 (Strengthening the Conclusion): As suggested, we have thoroughly revised the Conclusion section to make it more impactful. It now includes separate, clearly defined subsections for 'Recommendations for Future Research' and 'Specific Practical Applications', providing concrete and actionable guidance. We have also explicitly qualified our statement about the instrument being a "valuable tool" by integrating the necessary caveats regarding validation needs and implementation considerations, as advised.<br>These revisions were made in consideration of the complementary comments provided by Reviewer 2, ensuring a cohesive and comprehensive enhancement of the

N	Reviewer 1' Comments to Author	Author (s)' Response
		manuscript. We believe the manuscript is now much stronger and hope that the changes meet with the reviewer's approval.
	Novelty Assessment and Literature Comparison<br>1. Unique Contribution to the Literature<br>This study makes a significant and novel contribution to the intersection of disaster preparedness and chronic disease management. Through a comprehensive literature review, I identified that this represents the first validated disaster health literacy assessment tool specifically designed for diabetic patients. While several related instruments exist, none combine the disease-specific focus with disaster preparedness in a psychometrically validated format.<br><br>The existing literature includes general disaster literacy scales [1], diabetes-specific health literacy tools [2], and disaster preparedness guidelines for chronic diseases [3], but no instrument bridges these domains with the rigor demonstrated in this study. The German Disaster Health Literacy Scale [4] and Turkish Disaster Literacy Scale [5] focus on general populations, while comprehensive diabetes health literacy scales [6] address routine healthcare rather than emergency situations.	Thank you for recognizing the novel contribution of our study. We agree that this is the first validated disaster health literacy tool specifically designed for diabetic patients, and we appreciate your confirmation of its unique position in the literature.
	2. Comparison with Existing Instruments<br>General Disaster Health Literacy Tools<br>The most comparable instruments include the German Disaster Health Literacy Scale (Ewers et al., 2025) [4] and the Turkish Disaster Literacy Scale (Çalışkan & Üner, 2023) [5]. However, these tools differ fundamentally in their target population and scope:<br><br>German DIS-HL Scale: This 12-item instrument supplements the HLS19 for general disaster health literacy assessment. While methodologically sound, it lacks disease-specific content and does not address the unique challenges faced by diabetic patients during disasters. The validation approach, while appropriate, is less sophisticated than the MIRT methodology employed in the current study.<br><br>Turkish Disaster Literacy Scale: This comprehensive 61-item instrument covers four disaster phases (mitigation, preparedness, response, recovery) for the general population. While thorough, its length may limit practical utility, and it lacks the diabetes-specific focus	We appreciate your detailed comparison with existing instruments. Your analysis accurately highlights how our tool differs in its specific focus on diabetes during disasters, and we agree that this fills an important gap between general disaster literacy and chronic disease management.

N	Reviewer 1' Comments to Author	Author (s)' Response
	that makes the current study uniquely valuable.<br><br>Diabetes-Specific Health Literacy Instruments The Comprehensive Diabetes Health Literacy Scale (Lee et al., 2018) [6] represents the gold standard for diabetes health literacy assessment in routine healthcare settings. This 14-item instrument measures informational, numeracy, and communicative health literacy with strong psychometric properties (Cronbach's α = 0.91). However, it focuses entirely on routine diabetes management and lacks any disaster-specific content.<br><br>The current study's innovation lies in adapting health literacy assessment to emergency contexts while maintaining diabetes-specific relevance. The three-dimensional structure (risk perception literacy, medication-nutritional literacy, self-help literacy) represents a novel conceptualization that addresses the unique intersection of disaster preparedness and diabetes management.	
	3. Methodological Innovation The use of Multidimensional Item Response Theory (MIRT) for disaster health literacy assessment represents a methodological advancement over existing instruments. Most comparable tools rely on Classical Test Theory approaches, which may not adequately capture the complexity of multidimensional constructs. The MIRT approach allows for more nuanced understanding of item functioning and provides superior measurement precision across different ability levels.<br><br>The mixed-methods development approach, beginning with qualitative research [11, 12] and progressing to sophisticated quantitative validation, demonstrates methodological rigor that exceeds most existing instruments in this domain. The integration of scoping review findings with interview data provides a strong theoretical foundation that is often lacking in instrument development studies.	Thank you for noting the methodological innovation of using MIRT in our study. We believe this approach provides greater precision in measuring multidimensional constructs and hope it can inform future instrument development in disaster health literacy.
	4. Practical Significance and Gap Filling This instrument addresses a critical gap identified in disaster preparedness literature. Diabetic patients represent a particularly vulnerable population during disasters due to their dependence on medications, dietary requirements, and potential complications [7, 8]. Existing disaster preparedness resources for	We appreciate your acknowledgment of the practical significance of our instrument. Indeed, moving from educational checklists to a validated assessment tool represents an important step forward in supporting vulnerable populations during disasters.

N	Reviewer 1' Comments to Author	Author (s)' Response
	diabetic patients consist primarily of educational materials and checklists rather than validated assessment tools [9, 10].<br><br>The DHLQ enables systematic assessment of disaster preparedness knowledge and skills specific to diabetes management, facilitating targeted interventions and program evaluation. This capability is essential for emergency management agencies, healthcare systems, and public health organizations working to improve disaster resilience in vulnerable populations.	
	5. Limitations in Novelty Claims<br><br>While the study makes important contributions, some limitations in novelty should be acknowledged. The conceptual framework builds heavily on existing health literacy and disaster preparedness theories, and the dimensional structure shows similarities to established health literacy models. The innovation lies primarily in the specific application to diabetic patients in disaster contexts rather than fundamental theoretical advancement.<br><br>Additionally, while the instrument is novel in its specific focus, the broader concept of disaster health literacy has been explored in various forms. The authors appropriately acknowledge their previous work [11, 12] as foundational to this study, which demonstrates appropriate scholarly progression rather than entirely novel concept.	Thank you for this nuanced perspective. We agree that our innovation lies primarily in the specific application to diabetic patients in disaster contexts, and we have tempered our novelty claims accordingly in the revised manuscript.
	6. Future Research Implications<br>This study opens several important avenues for future research, including cross-cultural validation, predictive validity studies, and intervention effectiveness evaluation. The instrument provides a foundation for developing similar tools for other chronic conditions and for studying the relationship between disaster health literacy and actual emergency outcomes.<br><br>The methodological approach demonstrated here could serve as a model for developing disaster-specific health literacy instruments for other vulnerable populations, potentially leading to a family of related assessment tools that address various chronic conditions and special needs populations.	We appreciate your forward-looking comments. We agree that cross-cultural validation and expansion to other chronic conditions represent important next steps, and we hope our instrument can serve as a model for future research in this area.

N	Reviewer 2' Comments to Author<br><br>**Reviewer 2:**<br>**Thank you for the opportunity to improve our manuscript, we hope these revisions meet with your approval.**	**Author (s)' Response**
	Abstract<br>1. The phrase "content validity (CVI & CVI)" in the Methods section is a clear typo, likely intended as "CVI & CVR" (Content Validity Index and Content Validity Ratio), as detailed in the full Methods section.<br>2. In the Results section, "strong validity and reliability" is vague; please specify key metrics (e.g., Cronbach's alpha, acceptable fit indices) to show the validity and reliability obtained.	1. Thank you for your excellent attention to detail. You are absolutely correct; this was a typographical error. We appreciate your careful reading.<br>2. We thank the reviewer for this constructive feedback. The vague phrase "strong validity and reliability" has been removed from the abstract and replaced with specific, quantitative metrics.
	Introduction<br>The Introduction effectively sets the stage by outlining the global burden of diabetes, linking it to health literacy, highlighting vulnerabilities in disaster contexts, identifying a clear research gap (lack of a validated tool for disaster health literacy in diabetic patients), and stating the study's aim. However, I have some minor comments to strengthen this section:<br><br>1. The definition of "disaster health literacy" is embedded ("encompasses the skills and knowledge needed to effectively mitigate risks and respond to emergencies"), but I think it could be more explicitly defined or referenced earlier. Please consider adding a brief citation to a standard definition (e.g., from WHO or existing literature) to ground it theoretically, especially since this is central to the study.<br><br>2. There are a few run-on sentences and missing punctuation that could	1. We thank the reviewer for this insightful comment. The Introduction has been revised to include an explicit, cited definition of Disaster Health Literacy, which has been introduced in the earlier sections of the introduction.<br>2. Thank you for your valuable comment. The punctuation has been revised accordingly.<br><br>"post-disaster" the irrelevant sentence has been removed.<br><br>3. Thank you for your valuable feedback and insightful comment. We have revised the text to better contextualize our findings within the broader framework of disaster risks in low- and middle-income countries. The updated sentence now reads:<br>Research from Iran, a context of elevated disaster risk common to many low- and middle-income countries, reveals that approximately two-thirds of patients with diabetes or COPD lack sufficient disaster preparedness knowledge, highlighting a critical vulnerability that demands targeted intervention.<br><br>Please note that the reference numbering has changed due to the addition of new citations and subsequent reordering.

	be smoothed for better flow. For example:<br><br>"Furthermore, volunteer organizations often lack specialized resources for diabetic patients and strategies for maintaining proper medication storage conditions post-disaster Disaster health literacy encompasses...".<br>This appears to be a run-on; insert a dot or semicolon: "...post-disaster. Disaster health literacy encompasses..."<br>3. While the global burden is covered well, the section could acknowledge regional variations (e.g., higher disaster risks in low- and low-middle-income countries like Iran, where the study is based) to broaden its appeal. Additionally, the claim that "two-thirds of diabetic individuals lack adequate knowledge about disaster preparedness" (ref 7) is strong—ensure this reference is rob ust and generalizable, as it seems to be context-specific.	
	Methods<br>1. This section mentions sample sizes (e.g., 15 experts for content validity, 15 patients for face validity, 30 for test-retest, 570 for construct validity) but provides no information on how participants were selected, recruited, or sampled (e.g., convenience, purposive, random?). Inclusion/exclusion criteria (e.g., type 1 vs. type 2 diabetes, duration of illness, literacy levels) are absent, as is the study setting (e.g., specific clinics or regions in Iran). Demographics (e.g., age, gender, education) are not described, which is critical for assessing generalizability, especially in a culturally specific context like disaster preparedness. I recommend adding a subsection on "Participants and Sampling" with these details to enhance reproducibility.<br><br>2. While 570 for construct validity is reasonable for IRT/MIRT, there	1.We appreciate the reviewer's comment. In response, we have expanded the demographic description of participants to include additional variables such as diabetes duration, type of diabetes, comorbidities, obesity status, education level, and prior hazard experience (see paragraph one of Demographic statistics of participant's section, Table 3).<br><br>2.Thank you for your comment. The sample size of 570 participants was determined based on commonly recommended guidelines for both factor analysis and IRT modeling. For exploratory and confirmatory factor analysis, a sample of at least 5–10 participants per item is generally suggested; with the initial 37-item questionnaire, our sample meets this criterion (37 × 10 = 370). For IRT and MIRT analyses, larger samples improve parameter estimation stability, and previous studies suggest that 500–600 participants are sufficient for dichotomous item calibration in instruments of similar length. After item reduction, the final 30-item instrument still maintains an adequate subject-to-item ratio, ensuring robust estimation of item parameters and model fit. In this study, the 570 participants were selected using a convenience sampling approach (available participants), which allowed efficient data collection from the accessible. (see participants part of method section)

	is no rationale or power calculation provided.<br><br>3. Minor errors persist, e.g., "2 of medical librarians" should be "2 medical librarians"; "Ave/CVI-S" could be spelled out as "Scale-level Content Validity Index (S-CVI/Ave)" on first use.	3. Revised sentence in the manuscript: "…the Scale-level Content Validity Index (S-CVI/Ave) was calculated for the entire tool.
	Findings/Results<br>1. Use consistent terms (e.g., "Findings" vs. "Results" in abstract)<br><br>2. The Results flow logically but I suggest using subheadings (e.g., "Item Reduction," "Reliability," "Construct Validity") for better navigation.	1. We thank the reviewer for pointing out the inconsistency in terminology. We have revised the abstract to use the term "Results" consistently throughout the manuscript.<br><br>2. We sincerely thank the reviewer for the valuable suggestion to enhance the navigability of the Results section. In direct response to this feedback, we have incorporated the recommended subheadings—namely, 'Initial Item Pool Development,' 'Item Reduction,' 'Face Validity,' 'Reliability,' 'Construct Validity,' 'Factor Analysis,' 'Multidimensional Item Response Theory (MIRT) Analysis,' and 'Final Questionnaire Structure'—into the manuscript. We believe this revision significantly improves the clarity and flow for the reader, and we appreciate this constructive input.
	Discussion<br>1. I think the detailed comparison of IRT/MIRT vs. CTT (with refs 20-30) is informative but dominates the opening, reading more like a methods extension than interpretation. I suggest condensing this to focus on how MIRT enhanced your specific findings (e.g., precise domain extraction) and integrating it with results interpretation.<br><br>2. While limitations are explicitly stated (e.g., MIRT assumptions, retention of low-discrimination items, focus on self-help potentially overlooking contextual factors), they are somewhat understated and could be expanded for transparency. For instance, discuss how the Iranian sample limits generalizability to other cultural/disaster contexts (e.g., high-income vs. low-resource settings). Also, address potential biases in qualitative phases (e.g., expert selection) or the lack of criterion validity testing (e.g.,	1. We are grateful to Reviewer 2 for their thorough review and highly valuable feedback, which has been instrumental in refining the methodological narrative and transparency of our work.<br>Point 1 (Condensing IRT/MIRT Comparison): We thank the reviewer for this excellent suggestion. We have significantly condensed the technical comparison of CTT, IRT, and MIRT in the opening of the Discussion. The revised text now focuses directly on how the application of MIRT enhanced our specific findings, such as providing a more precise extraction of the multidimensional domains relevant to health literacy in disasters. This section is now better integrated with the interpretation of our results, moving away from a standalone methodological exposition.<br>2. Point 2 (Expanding Limitations for Transparency): We fully agree with the need for greater transparency regarding the study's limitations. In response, we have extensively expanded the 'Study Limitations' subsection within the Discussion. We now explicitly discuss the limited generalizability due to the Iranian sample, potential biases in qualitative phases (e.g., expert selection), and the lack of criterion validity testing, precisely as the reviewer recommended. This provides a much more balanced and critical perspective on our findings.<br>In making these revisions, we have also harmonized our responses with the constructive points raised by Reviewer 1, particularly in strengthening the practical implications and the conclusion. We hope that the revised discussion

	against existing health literacy tools).	and conclusion now fully address the reviewer's concerns and enhance the manuscript's overall rigor.

VERSION 2 - REVIEW

**Reviewer**	**1**
**Name**	**Hany, Mohamed**
**Affiliation**	**Alexandria University**
**Date**	**30-Oct-2025**
**COI**	

The revised manuscript demonstrates substantial improvements in methodological rigor, clarity of reporting, and transparency about limitations. No further revisions appear necessary based on the original reviewer comments.
